# Revealing the Transcriptional and Metabolic Characteristics of Sebocytes Based on the Donkey Cell Transcriptome Atlas

**DOI:** 10.1002/advs.202413819

**Published:** 2025-02-27

**Authors:** Yu Tian, Shuqin Liu, Hongtao Shi, Jianjun Li, Xinglong Wan, Yujiang Sun, Huayun Li, Ning Cao, Zhixi Feng, Teng Zhang, Junjie Wang, Wei Shen

**Affiliations:** ^1^ College of Animal Science and Technology Qingdao Agricultural University Qingdao 266109 China; ^2^ State Key Laboratory of Reproductive Regulation and Breeding of Grassland Livestock (R2BGL) College of Life Sciences Inner Mongolia University Hohhot 010070 China; ^3^ School of Science and Information Science Qingdao Agricultural University Qingdao 266109 China; ^4^ National Dezhou Donkey Original Breeding Farm Binzhou 251903 China; ^5^ Annoroad Gene Technology Beijing 100176 China

**Keywords:** cross‐species analysis, donkey, metabolomics, multi‐tissue single‐cell transcriptomic atlas, sebocyte

## Abstract

Worldwide, donkeys (*Equus asinus*) are valued for their meat and milk, and in China also for the medical value of their skin. Physiological characteristics are key to the donkey's adaptability, including their digestive, respiratory, and reproductive systems, which enable them to survive and work in a variety of environments. However, the understanding of donkey physiological characteristics at the cellular level remains poor. Thus, single‐cell transcriptome sequencing is used to construct a detailed transcriptional atlas based on 20 tissues from the Dezhou donkey (in total 84 cell types and 275 050 high quality cells) to perform an in‐depth investigation of molecular physiology. Cross‐species and cross‐tissue comparative analyses reveal *SOX10* to be an evolutionally conserved regulon in oligodendrocytes and illuminate the distinctive transcriptional patterns of donkey sebocytes. Moreover, through multispecies skin metabolomics, highly abundant, species‐specific metabolites in donkey skin are identified, such as arachidonic acid and gamma‐glutamylcysteine, and the pivotal role of sebocytes in donkey skin metabolism is highlighted. In summary, this work offers new insights into the unique metabolic patterns of donkey skin and provides a valuable resource for the conservation of donkey germplasm and the advancement of selective breeding programs.

## Graphical abstract

In this study, a comprehensive single‐cell transcriptome atlas of the Dezhou donkey, encompassing 20 tissues and 275 050 cells, is constructed. Through cross‐species and cross‐tissue analyses, distinct transcriptional patterns in donkey sebocytes are identified. Furthermore, multispecies skin metabolomics reveal species‐specific metabolites, emphasizing the critical role of sebocytes in shaping the unique metabolic profile of donkey skin.

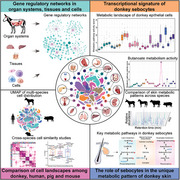

## 1 Introduction

The modern‐day donkey, which is commonly used as a livestock animal, is believed to have been domesticated from the Nubian donkey (*E. asinus africanus*) and the Somali donkey (*E. asinus somaliensis*).^[^ ^1^ ^]^ Following domestication, they were used for traction and transportation, playing a significant role in economic and cultural development throughout human history.^[^ ^2^ ^]^ Currently, 178 donkey breeds are recorded worldwide, but owing to the inadequate protection of germplasm resources and lack of standardized breeding practices, approximately a quarter of these breeds are at risk of extinction due to inbreeding and the indiscriminate introduction of foreign bloodlines.^[^ ^3^ ^]^ Therefore, there is an urgent need for the promotion of research programs focused on protecting donkey germplasm. More recently, the creation of a cell‐level species library has become an important conservation measure.

Recent transcriptome studies of donkeys have elucidated changes in gene expression and identified key functional genes involved in various biological processes, including muscle, skin, and testicular development,^[^ ^4^ ^]^ lactation,^[^ ^5^ ^]^ and oocyte maturation.^[^ ^6^ ^]^ However, these studies have predominantly used bulk RNA sequencing methods, which calculate an average number for gene expression levels across numerous cells and thus obscure gene expression heterogeneity at the single‐cell level. In contrast, single‐cell RNA sequencing provides a simultaneous and unbiased assessment of cell composition and cell type‐specific gene expression, capturing the intrinsic variability among individual cells. In addition, studies of multi‐tissue single‐cell atlases have revealed that cell type‐specific mechanisms and compositions play a crucial role in the development of complex traits and diseases in individuals.^[^ ^7^ ^]^ Therefore, by constructing a multi‐tissue single‐cell atlas for the donkey, it will be possible to identify key cell types and programs associated with economic traits, thereby facilitating precise breeding practices.

The traditional role of donkeys as draft animals has now evolved, because of the increasing global demand for products such as donkey milk, meat, and skin.^[^ ^8^ ^]^ Among these products, donkey skin holds a unique value. Donkey‐hide gelatin has been shown to improve blood profiles, assist blood clotting, strengthen the immune system, and provide anti‐aging benefits.^[^ ^9^ ^]^ Despite evidence confirming that donkey skin contains peptides that differ from those of horse, cattle, and pig skin, there remains a lack of detailed analysis regarding the molecular characteristics of donkey skin.^[^ ^10^ ^]^ Sebocytes are crucial functional cells in mammalian skin; they facilitate sebum production and also contribute to the regulation of sex hormone accumulation and metabolism within the skin.^[^ ^11^ ^]^ In addition, they coordinate cellular communication, influencing skin metabolism,^[^ ^12^ ^]^ and are involved in the initiation of innate immune responses.^[^ ^13^ ^]^ Furthermore, sebocytes play a role in regulating the skin's microecology and release antimicrobial peptides that enhance immune defenses.^[^ ^14^ ^]^ Therefore, the potential link between sebocytes and the unique medicinal properties of donkey skin warrants further investigation. The rapid development of cross‐tissue single‐cell analysis and nontargeted metabolomics analysis enables the systematic, high‐resolution transcriptional and metabolomic characterization of donkey skin through comparative studies across different tissues and species.^[^ ^15^ ^]^

The Dezhou donkey is characterized by high genetic diversity and low levels of hybridization among Chinese indigenous donkey breeds.^[^ ^16^ ^]^ This breed is larger in size and exhibits superior production traits, making it a promising candidate for breeding programs for skin, meat, and milk production.^[^ ^17^ ^]^ Moreover, the Dezhou donkey constitutes more than half of the total donkey population in China, providing a substantial genetic base for breed improvement.^[^ ^18^ ^]^ There are two strains of Dezhou donkeys: Wutou and Sanfen, with the Wutou strain demonstrating superior body size, growth rate, and slaughter traits compared to the Sanfen strain.^[^ ^4a^ ^]^ In addition, donkey‐hide gelatin extracted from the skin of the Wutou strain is reported to have higher medicinal value.^[^ ^19^ ^]^ In light of these factors, we constructed the first single‐cell transcriptome dataset of tissue types from adult Dezhou donkeys (Wutou strain) and established a Donkey Cell Atlas (DCA) database to facilitate the sharing and utilization of this valuable resource (https://egrb.qau.edu.cn/). Building on this foundation, we conducted a comprehensive comparative analysis at the cellular, tissue, and organ system levels to elucidate the molecular dynamics of donkeys. Furthermore, we integrated multispecies, multi‐tissue single‐cell atlases with metabolomic data to identify unique metabolic patterns in donkey sebocytes. This study provides a comprehensive overview of the molecular characteristics of donkeys and offers a valuable resource for the conservation of donkey germplasm and the advancement of selective breeding programs.

## 2 Results

### 2.1 Global Landscape of the Single‐Cell Transcriptomic Atlas of the Dezhou Donkey

**Figure 1:**
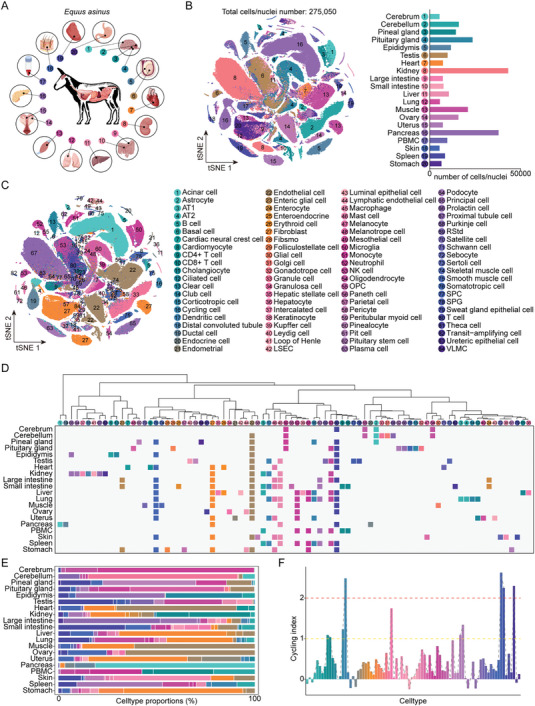
Generation of a cell atlas across 20 donkey tissue types. A) Schematic representation of donkey tissues analyzed in this study. B) Global clustering of all cells from the dataset colored by tissue (left) with tSNE map, and bar plots showing the number of high‐quality cells/nuclei profiled for each tissue (right). *n* = 275 050 individual cells/nuclei analyzed. C) tSNE visualization of all cell clusters colored by major cell types. A total of 84 cell clusters were identified in the dataset. Cell type annotation for all major clusters is provided in the right‐hand panel. AT1, alveolar type I cell; AT2, alveolar type II cell; Fibsmo, fibsmo cell; LSEC, liver sinusoidal endothelial cell; NK cell, natural killer cell; OPC, oligodendrocyte progenitor cell; Rstd, round spermatid; SPC, spermatocyte; SPG, spermatogonia; VLMC, vascular and leptomeningeal cell. D) Hierarchical clustering tree (top) showing the similarity (AUROC scores) among 84 donkey cell types and a heatmap (bottom) showing the various cell types present in different tissues. Cell type coloring is consistent with (C). E) The proportion of each cell type in different tissues. Cell type coloring is consistent with (C). F) Bar chart showing cell cycling index for each cell type. Cell type coloring is consistent with (C).

To generate a multi‐tissue single‐cell transcriptome reference atlas of the Dezhou donkey, we performed single‐cell and single‐nuclei transcriptome sequencing on 20 donkey tissues, including the cerebrum, cerebellum, pineal gland, pituitary gland, heart, peripheral blood mononuclear cells (PBMC), lung, kidney, spleen, liver, pancreas, stomach, large intestine, small intestine, muscle, skin, testis, epididymis, ovary, and uterus (**Figure** **1**A; Table S1, Supporting Information). Following rigorous quality control measures, a total of 275 050 cells were retained. Among these, kidney tissue yielded the largest number of single‐cell transcriptomic data, reaching 43 236, while skin provided the lowest number with 5448 cells (Figure 1B). T‐distributed stochastic neighbor embedding (tSNE) cluster analysis revealed the distinct distribution characteristics of cells within each tissue, indicating a high degree of tissue specificity at the transcriptional level. It is worth noting that most cells in the pineal gland and pituitary gland were clustered more closely, and most cells in the large intestine and small intestine were closer in distance, which may be related to their functional similarity (Figure S1, Supporting Information). Subsequently, we used uniform manifold approximation and projection (UMAP) to evaluate cell identities based on the expression patterns of specific cell type marker genes, along with performing Gene Ontology (GO) enrichment analysis (Figures S2–S4 and Tables S2–S4, Supporting Information). Results identified 84 major cell types, each by a sufficient number of unique molecular identifiers (UMI) and genes (Figure 1C; Figure S5, Supporting Information). Concurrently, the DCA website was developed based on the single‐cell transcriptome data generated in this study to facilitate resource sharing and utilization.

We performed a cluster analysis of the 84 identified donkey cell types based on their area under the receiver operator characteristic curve (AUROC) scores.^[^ ^20^ ^]^ The results indicated that functionally related cells exhibited greater similarity (Figure 1D). For instance, immune‐related cell types such as B cells, dendritic cells, kupffer cells, macrophages, mast cells, microglia, monocytes, neutrophils, NK cells, paneth cells, plasma cells, and T cells were observed to cluster together. In addition, we found that immune cells, endothelial cells, fibroblasts, and smooth muscle cells were present in most tissues and constituted a large proportion of the total cell count (Figure 1D,E). To identify cell states that were actively proliferating or quiescent, we calculated the cycling index for each cell type. Our analysis revealed that granulosa cells, immune cells, pituitary stem cells, spermatocytes (SPC), and spermatogonia (SPG), exhibited a high cycling index, indicative of active proliferation. In contrast, stromal cells, epithelial cells, and endocrine cells were predominantly in a quiescent state (Figure 1F).

### 2.2 Organ System Specific Expression Patterns in the Donkey

**Figure 2:**
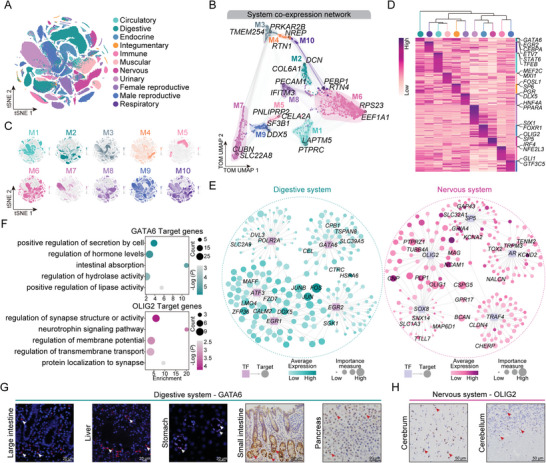
Molecular characterization of donkey organ systems. A) The tSNE visualization of global cell clustering from the dataset colored by organ system. B) TOM UMAP plot of the donkey organ system co‐expression network. Each node represents a single isoform, and edges represent co‐expression links between isoforms and module hub isoforms. Nodes are colored by co‐expression module assignment. C) UMAP visualization of expression patterns of module eigengenes. D) Heatmap illustrating the clustering analysis of 11 organ system specific TFs. Hierarchical clustering of biological systems according to RSSZ. Organ system categories coloring is consistent with (A). E) Integrated gene‐regulatory networks of the regulons in digestive and nervous systems. F) GO analysis of target genes of *GATA6* and *OLIG2*. G) Representative IHC staining of GATA6 in donkey large intestine, liver, stomach, small intestine, and pancreas. H) Representative IHC staining of OLIG2 in donkey cerebrum and cerebellum.

The 20 tissues under study were classified into 11 organ system categories to estimate the organ system‐specific genes involved in coordinating biological functions. As shown in **Figure** **2**A, the organs encompassed various systems: the circulatory system (heart and PBMC), digestive system (stomach, liver, large intestine, small intestine, and pancreas), endocrine system (pineal gland and pituitary gland), integumentary system (skin), immune system (spleen), muscular system (muscle), nervous system (cerebrum and cerebellum), urinary system (kidney), female reproductive system (ovary and uterus), male reproductive system (testis and epididymis), and respiratory system (lung). Subsequently, single‐cell weighted gene co‐expression network analysis (hdWGCNA) was performed with single‐cell datasets of different organs; 10 gene modules were generated and associated with different organ systems (Figure 2B; Figure S6A and Table S5, Supporting Information).^[^ ^21^ ^]^ By analyzing the expression patterns of co‐expressed modules in each organ system and comparing them with marker genes of donkey organ systems, the organ system‐specific enrichment in certain modules were identified. For example, modules 4 and 10 were enriched in the nervous system, module 5 in the digestive system, and module 7 in the urinary system. Other modules were associated with multiple organ systems, suggesting that these genes may regulate them (Figure 2C; Figure S6B,C, Supporting Information). GO enrichment analysis was used to identify the biological functions associated with co‐expression modules. The results revealed that modules 4 and 10 were enriched with genes related to synaptic transmission and neuronal development, module 5 contained genes involved in biological functions related to digestion and absorption, while module 7 was enriched in functions associated with organic acid metabolism and ion transport (Figure S6D,E, Supporting Information). The findings suggested that the nervous, digestive, and urinary systems had unique functional gene regulatory networks distinct from those of other organ systems. Interestingly, we observed a significant overlap of genes between module 3 and both the nervous and endocrine systems, despite the low expression levels of these genes in both organ systems. KEGG enrichment analysis revealed that these genes did not correspond to classical pathways for neural regulation (Figure S6F, Supporting Information).^[^ ^22^ ^]^ However, existing studies indicate that these pathways are activated in response to external stimuli or pathological conditions to regulate neural‐related biological processes. These findings suggest that the genes in module 3 may be involved in specialized regulatory processes, potentially contributing to adaptive or stress‐related responses under specific conditions.

Transcription factors (TFs) play pivotal roles in cell type determination, developmental patterning, and the regulation of specific pathways.^[^ ^23^ ^]^ Through SCENIC analysis, we were able to identify key TFs that govern distinct organ systems in the donkey.^[^ ^24^ ^]^ Based on Z‐score normalized regulon specificity scores (RSSZ) analysis, 295 TFs specific to different organ system classes were identified.^[^ ^25^ ^]^ Among the different organ systems, the urinary system exhibited the highest number of system‐specific TFs, while the digestive system demonstrated comparatively fewer (Figure 2D). In addition, given the unique transcription patterns exhibited by the nervous and digestive systems, we constructed specific gene regulatory networks of these systems (Figure 2E). Within the gene regulatory network of the digestive system, we observed that GATA6 regulated target genes involved in digestive enzyme secretion (*CEL*,^[^ ^26^ ^]^ *CTRC* ^[^ ^27^ ^]^), energy metabolism (*GLS2*,^[^ ^28^ ^]^ *LMO4* ^[^ ^29^ ^]^), and nutrient absorption (*CPB1*,^[^ ^30^ ^]^ *SLC39A5* ^[^ ^31^ ^]^). In the nervous system, highly active TFs regulate target genes associated with neuronal development (*TUBB4A* ^[^ ^32^ ^]^), and neuronal morphogenesis (*NCAM1* ^[^ ^33^ ^]^). To explore whether TFs performed the same functions within each organ system, we extracted target genes for highly active TFs *GATA6* in the digestive system and *OLIG2* in the nervous system, and GO enrichment analysis on these genes was conducted. *GATA6*‐regulated target genes were involved in biological functions related to intestinal absorption and digestive enzyme activity, while *OLIG2* target genes were associated with neuronal activity (Figure 2F). At the same time, we examined the expression characteristics of GATA6 in the large intestine, small intestine, liver, stomach, and pancreas, as well as OLIG2 in the cerebrum and cerebellum (Figure 2G,H). The results demonstrated that GATA6 and OLIG2 were ubiquitously expressed across various tissues within their respective organ systems.

### 2.3 Tissue Category‐Specific Transcription Factor Regulatory Network in the Donkey

**Figure 3:**
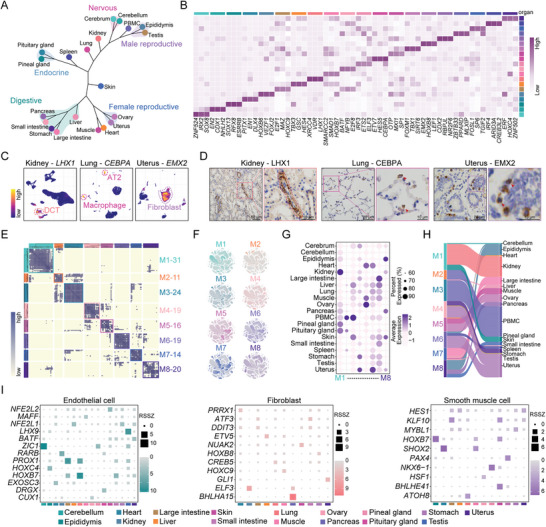
Characterization of transcription factor networks across organ category. A) Dendrogram showing hierarchical clustering results for different tissues. B) Heatmap depicting the three tissue‐specific TFs with the highest RSSZ rankings across 20 tissues. Tissue type coloring is consistent with (A). C) UMAP visualization of the expression of *LHX1* in kidney, *CEBPA* in lung, and *EMX2* in uterine tissues. D) Representative IHC staining for LHX1 in kidney, CEBPA in lung and EMX2 in uterine tissues. E) Identification of eight TF modules based on the regulon CSI of the cell landscape in the donkey. F) UMAP illustrating the average AUCell score distribution for different regulon modules. G) Bubble chart showing the expression levels of TF modules in different tissues. H) Sankey plot showing the connectivity between regulon modules (modules 1–8) and donkey cell categories. I) Heatmap displaying the TFs RSSZ for endothelial cells, fibroblasts, and smooth muscle cells across various donkey tissues.

Next, the transcriptional similarity of all tissues was depicted by using gene expression trees. Among the 20 tissues, the digestive, endocrine, reproductive, and nervous systems exhibited high similarity within their respective organ systems. Notably, the gene expression profiles of the heart and muscle also demonstrated significant similarity (**Figure** **3**A). These findings suggest that functionally similar tissues possess closely related transcriptional profiles. To identify core driver TFs across different tissues, we used RSSZ to pinpoint highly tissue‐specific TFs (Figure 3B). Simultaneously, we focused on the three most active TFs: *LHX1*, *CEBPA*, and *EMX2*. UMAP plots of various tissues revealed that *LHX1* was highly expressed in the distal convoluted tubule (DCT) of kidney tissues, *CEBPA* in alveolar type 2 cells (AT2) and macrophages of the lungs, and *EMX2* in uterine fibroblasts (Figure 3C). We also conducted immunohistochemistry (IHC) analysis and further confirmed their location of expression in tissues (Figure 3D).

TFs frequently coordinate gene expression in a combinatorial manner. We identified eight TF regulon modules in donkeys using a combination of the connectivity specificity index (CSI) (Figure 3E; Table S6, Supporting Information).^[^ ^25^ ^]^ To identify tissue category‐specific modules, the activity of gene sets within different modules was scored using AUCell. The results revealed that the TF regulatory submodule exhibited a tissue category‐specific expression pattern. For instance, module 1 was highly expressed in the kidney, modules 2 and 3 were predominantly expressed in PBMC, modules 4 and 6 were actively expressed in the uterus, module 5 was highly active in the ovary, module 7 was primarily expressed in the large intestine, and module 8 showed high activity in the epididymis (Figure 3F,G). Subsequently, we quantified the average expression levels of TFs in each module across different tissues and mapped the expression of TFs within each module to their respective tissues (Figure 3H). Simultaneously, we performed GO enrichment analysis to characterize the biological functions associated with the TF regulators within each module. Notably, module 1 was enriched in functional terms such as “monoatomic cation transport” and “renal system development,” indicating that it primarily maintains kidney function homeostasis. For modules 2 and 3, the functional analysis identified terms related to the “activation of immune response,” suggesting that these modules are mainly involved in immune regulation. Module 5 was enriched in terms of “reproductive system development,” indicating its role in ovarian development. Module 7 was enriched in terms of intestinal function (Figure S7A, Supporting Information). These findings highlighted that our systematic identification of donkey TFs successfully identified tissue category‐specific TF regulon modules.

We also conducted cross‐tissue TF network predictions for three cell types prevalent in different tissues: endothelial cells, fibroblasts, and smooth muscle cells. The results demonstrated that TF activity was distinctly tissue‐specific. For example, *LHX9* showed high activity in testicular endothelial cells, while *ZIC1* was specifically highly expressed in cerebellar endothelial cells; meanwhile, *BHLHA15* was significantly active in pancreatic fibroblasts. Similarly, in smooth muscle cells, *HOXB7* and *SHOX2* exhibited high activity in the epididymis and heart, respectively (Figure 3I). Finally, we inferred cell category‐specific TF regulatory networks (Figure S7B, Supporting Information). We observed that *ALX3* acted as a specific TF regulator in folliculostellate cells, while *NHLH2* showed high activity in Golgi cells. Notably, we found that the proportion of tissue‐specific cell type‐specific transcription factors (CSTFs) relative to tissue‐shared CSTFs was higher in most tissues. This finding indicated that the regulatory networks of tissue‐specific TFs in these tissues were predominantly driven by tissue‐specific cell type (Figure S8A, Supporting Information). In addition, we identified the tissue‐specific TFs in each tissue that overlapped with tissue‐specific CSTFs, highlighting their potential roles as key regulators driving the unique functional characteristics of tissues (Figure S8B, Supporting Information).

### 2.4 Cross‐Species Cell Type Similarity Analysis Revealed Transcriptional Signatures of Donkey Sebocytes

**Figure 4:**
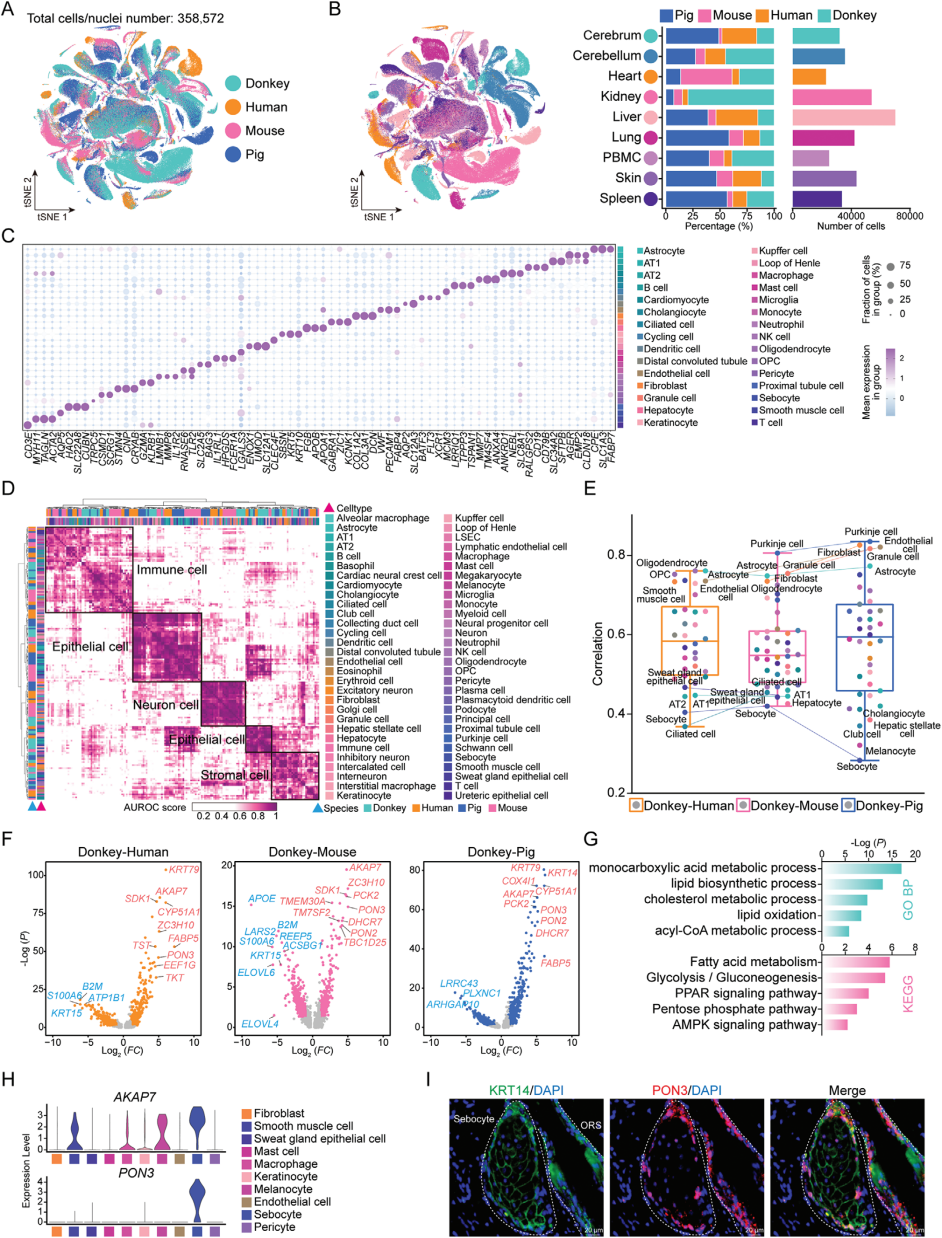
Comparative analysis of multispecies cellular landscapes. A) A tSNE map of global cell clustering from the dataset colored by species. *n* = 358 872 individual cells/nuclei analyzed. B) tSNE visualization of global cell clustering from the dataset colored by tissue (left), and bar plots showing number and percentage of cell types for each species (right). C) Dot plot showing representative marker genes of different cell types. Dot size is proportional to the fraction of cells expressing specific genes. Color intensity corresponds to the relative expression levels of genes. D) Correlation of orthologous gene expression between human, donkey, pig, and mouse pseudo‐cell types based on the AUROC scores. The AUROC scores were calculated by MetaNeighbor to measure the similarity between different cell types. E) Box plots based on Spearman's correlation of average gene expression for a specific cell type between two different species. Cell type coloring is consistent with (D). F) Volcano plot displaying differentially expressed genes of sebocytes between the donkey and other species. G) Results of GO and KEGG enrichment analysis of highly expressed genes in donkey sebocytes. H) Violin plots visualizing the expression of *AKAP7* and *PON3* across various cell types in donkey skin. I) Representative images of PON3 staining (red) in donkey sebocytes. Nucleus was counterstained with DAPI (blue). Sebocytes were labeled with KRT14 (green).

Cross‐species comparative analysis of single‐cell transcriptome data is essential for identifying donkey‐specific molecules and elucidating the regulatory mechanisms underlying complex phenotypes. Humans, pigs, and mice are pivotal mammalian models in the fields of disease research, developmental biology, and agriculture. These species have well‐established single‐cell transcriptome datasets encompassing various tissues, accompanied by publicly validated analytical frameworks. Here, we integrated single‐cell transcriptome data from nine major tissues (cerebrum, cerebellum, heart, kidney, liver, lung, PBMC, skin, and spleen) from humans (72 475 cells), donkeys (110 864 cells), pigs (133 842 cells), and mice (41 391 cells). The integrated cell atlas comprises transcriptome data for 358 572 high‐quality cells, with cell counts ranging from 70 088 in the liver to 22 796 in the heart (**Figure** **4**A,B). Next, the cell type‐specific marker genes common in the four species were identified through differential analysis. The expression patterns of these representative marker genes were generally consistent across the species (Figure 4C). To further investigate whether these cell types had conserved TFs across species, we identified cell class‐specific TFs in each: 342 in donkeys, 285 in humans, 279 in mice, and 390 in pigs. We found that some of these TFs exhibited similar activity characteristics across all four species. Specifically, *HNF4A*, *FOXA1*, and *FOXA2* were highly active in epithelial cells; *EOMES* and *NFE2* were highly expressed in immune cells; *SOX10* and *NEUROD1* showed characteristic expression in neuronal cells; and *ERG* was more highly expressed in stromal cells (Figure S9A, Supporting Information). Notably, based on regulator activity scores and RSSZ, *SOX10* may serve as a key TF in oligodendrocytes across the four species (Figure S9B, Supporting Information). These target genes exhibited higher expression levels in oligodendrocytes of various species and were significantly enriched in neurodevelopment‐related functions such as “oligodendrocyte differentiation” (Figure S9C,D, Supporting Information). In summary, these results identified conserved transcriptional signatures among the major cell classes across the four species.

To further highlight the divergence of donkey cell types from those of other species, we utilized MetaNeighbor to analyze the similarities among 60 cell types across four species.^[^ ^20^ ^]^ As shown in Figure 4D, immune cells, neuron cells, epithelial cells, and stromal cells exhibited conserved gene expression patterns across the four species. Notably, correlation analysis of donkey single‐cell data with that of the other three species revealed a low correlation for epithelial cells. This may be attributed to the fact that most epithelial cells are terminally differentiated functional cells, which are more closely associated with specific functional characteristics in each species. In addition, we found that the gene expression pattern of donkey sebocytes showed the lowest correlation with those of the other three species (Figure 4E; Table S7, Supporting Information). To uncover the gene expression characteristics of sebocytes, we identified their differentially expressed genes between donkeys and the other species (Figure 4F). Our analyses revealed that the expression levels of *AKAP7* and *PON3* in donkey sebocytes were significantly higher compared to those in the other three species. Simultaneously, we performed GO and KEGG enrichment analyses on genes highly expressed in donkey sebocytes. The results indicated that these genes are involved in biological functions, such as “fatty acid metabolism” and “glycolysis/gluconeogenesis” (Figure 4G). Furthermore, we performed GO and KEGG enrichment analyses of the differentially expressed genes in sebocytes between donkeys and other species. These analyses highlighted that donkey sebocytes exhibited higher expression levels of genes related to energy metabolism and lipid metabolism compared to other species, while genes associated with protein metabolism were more highly expressed in sebocytes of other species (Figure S10A,B, Supporting Information). This observation may highlight the enhanced energy and lipid metabolism of donkey sebocytes. Notably, we observed that the expression levels of *AKAP7* and *PON3* in donkey sebocytes were significantly higher than in other cell types, with *PON3* being particularly prominent (Figure 4H). Further verification using immunofluorescence (IF) confirmed that PON3 served as a specific marker for donkey sebocytes (Figure 4I).

### 2.5 Cross‐Tissue Transcriptional Signatures of Epithelial Cells

**Figure 5:**
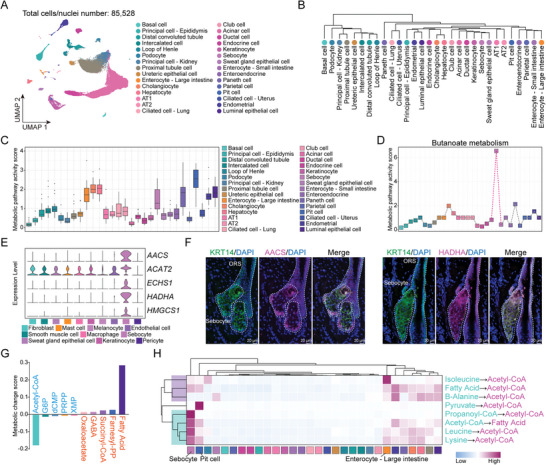
Transcriptional characterization of inter‐tissue epithelial cells. A) UMAP plot showing clustering results of epithelial cells colored by cell type. B) Dendrogram depicting the hierarchical clustering of various epithelial cell types. C) Distribution of metabolic pathway activity in different epithelial cell types in the donkey single‐cell transcriptome dataset. D) Butanoate metabolism activity score in different epithelial cell types. Cell type coloring is consistent with (C). E) Violin plots visualizing the expression of *AACS*, *ACAT2*, *ECHS1*, *HADHA*, and *HMGCS1* across various cell types in donkey skin. F) Representative images of AACS and HADHA staining in donkey sebocytes. Nuclei were counterstained with DAPI (blue). Sebocytes were labeled with KRT14 (green). G) The top accumulated and depleted metabolites predicted in sebocytes. The *y*‐axis denotes metabolism stress level (or level of accumulation and depletion), where a positive value represents accumulation and a negative value represents depletion. The *x*‐axis denotes metabolites in a decreasing order of accumulation level. H) Heatmap showing predicted cell‐wise flux results for acetyl‐CoA‐related metabolic reactions in different epithelial cell types. Cell type coloring is consistent with (C).

Given that various epithelial cells in donkeys exhibited distinct transcriptional profiles in cross‐species comparative analyses, we extracted 85 528 epithelial cells from ten different donkey tissues for a cross‐tissue study to examine epithelial cell heterogeneity (**Figure** **5**A). After performing differential expression analysis, we identified unique gene expression patterns in various epithelial cells (Table S8, Supporting Information). In addition, we hierarchically clustered each epithelial cell type based on the expression matrix of differentially expressed genes. The results showed that most epithelial cells were clustered into tissue‐specific categories, such as kidneys and skin. Moreover, functionally similar cells, such as ciliated cells and intestinal epithelial cells, exhibited high similarity in their gene expression profiles (Figure 5B). UMAP analysis results of epithelial cells also indicated that epithelial cells within the same tissue were more tightly clustered compared to those from different tissues (Figure S11A, Supporting Information). Next, we performed GO analysis on the differential gene sets of various epithelial cell types to uncover their unique biological functions (Table S9, Supporting Information). Notably, the top three GO terms for different types of epithelial cells, ranked by *p*‐value, indicated that most cells exhibited metabolism‐related biological functions (Figure S11B, Supporting Information).

To construct the metabolic landscape of each epithelial cell type across different donkey tissues, we compared metabolic gene expression profiles using a single‐cell metabolic landscape pipeline (see the Experimental Section).^[^ ^34^ ^]^ By calculating the metabolic pathway activity score, we observed that cholangiocytes, endometrial cells, hepatocytes, enterocytes–large intestine, luminal epithelial cells, Paneth cells, pit cells, and sebocytes all exhibited high metabolic activity (Figure 5C). In addition, we identified metabolic signatures unique to various epithelial cell types across ten different tissues (Figures S12–S14, Supporting Information). Notably, butanoate metabolism was significantly more active in sebocytes compared to other epithelial cell types (Figure 5D). The key genes in the butanoate metabolism pathway were highly expressed in sebocytes, with *AACS* and *HADHA* showing particularly elevated expression levels (Figure 5E). Furthermore, IF analysis of donkey skin clearly identified the expression of AACS and HADHA in sebocytes (Figure 5F). We also used single‐cell flux estimation analysis (scFEA) to simulate the metabolic flux in sebocytes.^[^ ^35^ ^]^ As shown in Figure 5G, acetyl‐CoA is consumed in large quantities in sebocytes, with a significant accumulation of fatty acids. In addition, we quantified the flux of metabolic modules related to the consumption and generation of acetyl‐CoA in various epithelial cell types. Sebocytes, in particular, tend to consume propionyl‐CoA, leucine, and lysine to generate acetyl‐CoA. Furthermore, the metabolic flux from acetyl‐CoA to fatty acid generation in sebocytes is significantly higher than in other epithelial cells (Figure 5H). Collectively, our results indicated that donkey sebocytes primarily generated fatty acids through acetyl‐CoA produced via the butyrate metabolic pathway, thereby maintaining sebocyte function.

### 2.6 Multispecies Metabolomics Analysis Unveiled the Unique Metabolic Characteristics of Donkey Skin

**Figure 6:**
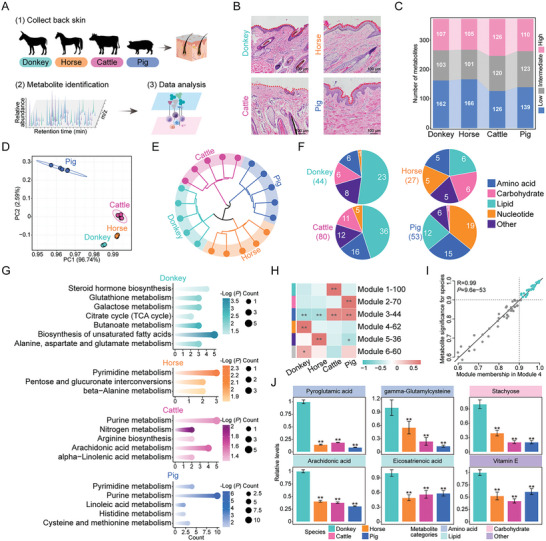
Metabolic characteristics of skin in donkeys, horses, cattle, and pigs. A) Flowchart of experimental design for obtaining metabolome data from donkey, horse, cattle, and pig skin. B) HE staining of skin sections from donkeys, horses, cattle, and pigs. C) Alluvial plots based on high, intermediate, and low metabolite levels in the skin of four species. D) PCA analysis of metabolome samples from donkeys, horses, cattle, and pigs. Points marked with the same color represent five replicates of the same species. E) Dendrogram showing hierarchical clustering results of skin metabolome data of four species. F) Pie plot showing the percentage of metabolite categories for identified metabolites in the skin of the four species. G) KEGG analysis of four species‐specific high‐abundance metabolites. H) Heatmap showing the correlation between different species and functional modules identified by WGCNA. I) Scatter plot showing the relationship between module 4 membership and metabolite significance. J) Relative levels of representative metabolites associated with metabolite categories in the skin of different species.

To further elucidate the unique metabolic pattern of donkey skin, we conducted metabolomic analysis on the dorsal skin of donkey, horse, cattle, and pig species commonly used in gelatin products (**Figure** **6**A). First, histological examination of the dorsal skin sections revealed the notable differences among the studied species. In particular, donkey skin exhibited a significantly higher density of collagen fibers compared to that of other species (Figure 6B). Next, following component annotation, a total of 372 distinct metabolites in the dorsal skin of the four species were identified (Table S10, Supporting Information). Furthermore, the metabolites in the four species were categorized into three groups based on metabolite abundance: high, intermediate, and low (see the Experimental Section). The metabolic contents of the four species were significantly different, with pigs and cattle exhibiting significantly more high‐content metabolites in their dorsal skin compared to the donkey and horse (Figure 6C). In addition, we observed significant differences in the distribution patterns of metabolite categories across the four species at different abundance levels (Figure S15A, Supporting Information). The outcomes of principal component analysis (PCA) and hierarchical cluster analysis further elucidated distinct metabolic profiles among the four species. Specifically, the metabolite levels of dorsal skin in pigs exhibited significant deviation from those observed in the other three species (Figure 6D). Furthermore, the metabolic patterns of donkey and horse samples displayed a greater degree of similarity within the studied cohort (Figure 6E).

Orthogonal partial least squares‐discriminant analysis (OPLS‐DA) is often employed to identify metabolites that differ significantly between groups. In our study, based on OPLS‐DA pairwise comparisons among the four species, a total of 204 differential metabolites were identified (Figure S15B,C, Supporting Information). Among these metabolites, 44 were significantly higher in donkey skin compared to the other species, and 23 of these were lipids. In horse skin, 27 characteristic metabolites were identified. In cattle skin, 80 characteristic metabolites were detected, the majority of which were lipids. In pig skin, 53 characteristic metabolites were identified, the majority of which were amino acid and nucleotide class metabolites (Figure 6F). Next, we utilized KEGG to map the characteristic metabolites of skin to metabolic pathways in each species, aiming to reveal the unique metabolic activities of different species (Figure 6G). Among these, the metabolic activities of pathways such as “Biosynthesis of unsaturated fatty acids,” “Steroid hormone biosynthesis,” “TCA cycle,” “Butanoate metabolism,” and “Glutathione metabolism” were significantly higher in donkey skin compared to that in horses, cattle, and pigs. In contrast, we observed greater activity in nucleotide metabolic pathway in the skin of horses, cattle, and pigs compared to donkeys. To provide more detailed annotations of metabolic functions, we further enriched the characteristic metabolites of each species using the Reactome database.^[^ ^36^ ^]^ Analyses revealed that, in addition to the metabolic pathways enriched in KEGG database, donkey skin exhibited significant enrichment in pathways related to ketone body metabolism, arachidonic acid metabolism, and vitamin E. Furthermore, biological processes associated with phospholipid metabolism were notably enriched in cattle skin (Figure S16, Supporting Information). We also constructed a metabolite co‐expression network using WGCNA and identified six co‐expression modules, with module 4 showing a significant association with donkey skin (Figure 6H).^[^ ^37^ ^]^ Based on module 4 membership and metabolite significance, we screened the characteristic metabolites of donkey skin (Figure 6I). Among these metabolites, pyroglutamic acid and gamma‐glutamylcysteine, both involved in amino acid metabolism, were highly abundant in donkey skin. In addition, stachyose, a metabolite in carbohydrate metabolism, was significantly more abundant in donkey skin when compared to the other three species. In lipid metabolism, arachidonic acid and eicosatrienoic acid were also highly abundant in donkey skin. Moreover, vitamin E levels were significantly higher in donkey skin than in other species (Figure 6J). In summary, these data highlighted that donkey skin possesses distinctive metabolic characteristics, particularly in lipid metabolism.

### 2.7 Sebocytes Were Shown to Be Involved in Shaping the Unique Metabolic Pattern of Donkey Skin

**Figure 7:**
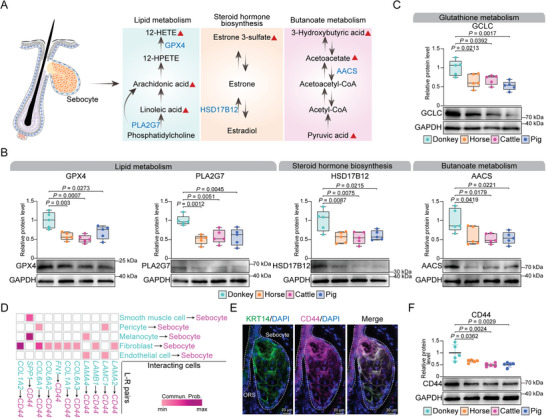
Cross‐species comparison reveals expression characteristics of key metabolic genes in donkey skin. A) Schematic diagram illustrating lipid metabolism, steroid hormone biosynthesis, and butyrate metabolism in sebocytes. Metabolites that are uniquely abundant in donkey skin are indicated by red‐filled triangles. Metabolites are labeled in black, while genes specifically highly expressed in sebocytes are labeled in blue. B) The protein levels of GPX4, PLA2G7, HSD17B12, and AACS in donkey, horse, cattle, and pig skin were detected. GAPDH was used as an internal control to calculate the relative protein levels (*n* = 5 biologically independent replicates). C) Detection of GCLC protein levels in donkey, horse, cattle, and pig skin. GAPDH was used as an internal control to calculate relative protein level (*n* = 5 biologically independent replicates). D) Important ligand–receptor pairs between sebocytes and other cell types in donkey skin. E) Representative images of CD44 staining in donkey sebocytes. Nuclei were counterstained with DAPI (blue). Sebocytes were labeled with KRT14 (green). F) Detection of CD44 protein levels in donkey, horse, cattle, and pig skin. GAPDH was used as an internal control to calculate relative protein levels (*n* = 5 biologically independent replicates).

Sebocytes are crucial for lipid and steroid metabolism in the skin.^[^ ^38^ ^]^ Our single‐cell transcriptome data of donkey skin revealed that sebocytes exhibit significantly higher activity in “Fatty acid elongation,” “Butanoate metabolism,” and “TCA cycle” compared to other cell types (Figure S13D, Supporting Information). These findings suggest that the unique metabolic activities observed in donkey skin are closely associated with sebocytes. To further elucidate the metabolic regulation of the sebocyte environment in donkey skin, we integrated those genes specifically expressed in sebocytes with the key metabolites in donkey skin. We noted that PLA2G7 in sebocytes was involved in the synthesis of linoleic acid and arachidonic acid, while GPX4 regulated the metabolism of 12‐HETE. In addition, HSD17B12 was implicated in the conversion of estradiol to estrone 3‐sulfate, and AACS regulated the conversion of acetoacetate to acetoacetyl‐CoA (**Figure** **7**A).^[^ ^39^ ^]^ Subsequently, protein expression levels of GPX4, PLA2G7, HSD17B12, and AACS in the skin of donkeys, horses, cattle, and pigs were detected (Figure 7B). The results showed that the relative abundance of these proteins was significantly higher in donkeys than in other species. In addition, we observed that the expression level of GCLC, the rate‐limiting enzyme involved in the synthesis of gamma‐glutamylcysteine in glutathione metabolism, was markedly higher in donkey skin (Figure 7C). Notably, the ligand‐receptor network constructed for the donkey skin cell population revealed that donkey sebocytes were regulated by ligand signals from smooth muscle cells, pericytes, melanocytes, fibroblasts, and endothelial cells, with *CD44* identified as a key receptor (Figure 7D; Figure S17A–C, Supporting Information).^[^ ^40^ ^]^ Further analysis of the effects of *CD44* on target genes associated with intracellular communication processes revealed that the 41 target genes regulated by *CD44*‐mediated signaling in sebocytes were predominantly involved in biological functions related to cell metabolism. This observation underscored the pivotal role of *CD44* as a key receptor in regulating sebocyte metabolic processes (Figure S17D,E, Supporting Information).^[^ ^41^ ^]^ IF staining confirmed the high expression of CD44 in donkey sebocytes (Figure 7E). Interestingly, we also observed that the protein level of CD44 in donkey skin was significantly higher than that of horse, cattle, and pig skin (Figure 7F). This result underscored the significant association between CD44 and the distinct metabolic processes in donkey sebocytes. Collectively, these findings suggest that sebocytes play a significant role in shaping the unique metabolic pattern of donkey skin.

## 3 Discussion

Donkeys are an important livestock resource, valued for their meat, milk, and various medical uses. Despite the rising market demand for donkey products, there has been no corresponding increase in the donkey population. On the contrary, the number of donkeys in China has plummeted from 9.44 million in 1996 to 1.46 million in 2023,^[^ ^42^ ^]^ posing a significant threat to the quantity and quality of donkey germplasm resources. Currently, the Dezhou donkey represents over 57% of the total donkey population in China, primarily due to its rapid growth rate, superior production performance, and stable genetic traits.^[^ ^18^ ^]^ Notably, the Dezhou donkey (Wutou strain) grows faster than other breeds, and the donkey‐hide gelatin extracted from its skin possesses higher medicinal value.^[^ ^4^, ^19^ ^]^ In addition, Wang et al. successfully de novo assembled a chromosome‐level reference genome for the Dezhou donkey, thereby establishing a crucial foundation for molecular research on this breed.^[^ ^1^ ^]^ Consequently, the current study developed a multi‐tissue single‐cell reference map for the Dezhou donkey (Wutou strain), conducted a comprehensive analysis across various tissues and organ systems, and highlighted the critical role of donkey sebocytes in shaping the unique metabolic profile of donkey skin.

The current study provides a whole transcriptome landscape of 20 major tissues from adult Dezhou donkeys in single cell resolution, encompassing 11 principal organ systems. In total, 84 cell types were identified and annotated, including rare ones like Purkinje cells and enteroendocrine cells. Notably, we observed that these 84 cell types were clustered based on functional similarities, a finding consistent with Chen et al.’s conclusions in their multi‐tissue cell atlas of pigs.^[^ ^7a^ ^]^ Our results further indicated that TFs not only regulated specific cell types but also exhibited high transcriptomic activity with tissue‐ and organ system‐specific characteristics. For instance, *GATA6* and *OLIG2* were identified as organ system‐specific TFs in the donkey digestive and nervous systems, respectively. Research in humans and mice has underscored the critical roles of *GATA6* in intestinal crypt cells, secretory cells, Paneth cells, cholangiocytes, and pancreatic β cells,^[^ ^43^ ^]^ as well as *OLIG2* in glial cell populations within the cerebrum and cerebellum.^[^ ^44^ ^]^ These findings suggest that *GATA6* may serve as a key molecular target for enhancing digestive efficiency and maintaining gastrointestinal health in donkeys, while *OLIG2* may be crucial for sustaining neural function homeostasis.

To further elucidate the conservation and distinct characteristics between donkeys and other mammals, we performed a cross‐species multi‐tissue single‐cell atlas analysis. Our study demonstrated that gene expression patterns of major cell types, such as immune, matrix, neuronal, epithelial, and endothelial cells, were conserved in donkeys, which corroborates previous reports in other vertebrates.^[^ ^45^ ^]^ Interestingly, we noted that donkey sebocytes exhibited lower correlations in comparative analyses with those from humans, mice, and pigs. Specifically, *PON3* was found to be highly expressed in donkey sebocytes. As reported, PON3, an antioxidant hydrolase, inhibits mitochondrial superoxide production, thereby exerting antioxidant and anti‐apoptotic effects.^[^ ^46^ ^]^ In mice, deficiency of *Pon3* impairs mitochondrial function and disrupts lipid metabolism.^[^ ^47^ ^]^ This suggests that *PON3* may be a key factor in sebocytes; however, whether it plays a role in distinctive energy and lipid metabolism still needs further investigation in donkeys.

Donkey‐hide gelatin is recognized for its unique medicinal value, which we speculate is linked to the distinct metabolic components of donkey skin. Our study revealed that donkey skin contains significantly higher levels of metabolites, such as gamma‐glutamylcysteine and arachidonic acid, compared to skin from horses, cattle, and pigs. Furthermore, the protein levels of GCLC (the rate‐limiting enzyme in gamma‐Glutamylcysteine synthesis) and PLA2G7 (the rate‐limiting enzyme in arachidonic acid synthesis) were also markedly higher in donkey skin relative to other species. Among them, gamma‐glutamylcysteine, a direct precursor of glutathione, is crucial;^[^ ^48^ ^]^ glutathione plays a significant role in redox signaling, cell proliferation, apoptosis, and immune function;^[^ ^49^ ^]^ however, it is not readily absorbed.^[^ ^50^ ^]^ In contrast, gamma‐glutamylcysteine can be efficiently transported across cell membranes, facilitating intracellular glutathione synthesis.^[^ ^51^ ^]^ Numerous studies highlight the remarkable antioxidant and anti‐inflammatory properties of gamma‐glutamylcysteine, underscoring its potential therapeutic benefits.^[^ ^52^ ^]^ In addition, arachidonic acid, a critical metabolite of sebocytes,^[^ ^11^ ^]^ plays a vital role in numerous physiological processes, including inflammation resolution,^[^ ^53^ ^]^ diabetes prevention,^[^ ^54^ ^]^ cholesterol regulation,^[^ ^55^ ^]^ improvement of the intracellular environment, and the regulation of cell renewal.^[^ ^56^ ^]^ Cheng et al. demonstrated that supplementation with Ejiao peptide‐iron chelates significantly elevated plasma arachidonic acid levels in mice with iron deficiency anemia.^[^ ^57^ ^]^ These findings suggest that the high levels of gamma‐glutamylcysteine and arachidonic acid in donkey skin may greatly contribute to the medicinal value of donkey‐hide gelatin. Another noteworthy finding is the significantly higher expression of CD44 in donkey sebocytes compared to those in horses, cattle, and pigs. CD44, a cell surface glycoprotein, has been identified as a key regulator in the metabolic switch between lactate glycolysis and oxidative phosphorylation in cancer cells.^[^ ^58^ ^]^ Jiang et al. demonstrated that *CD44* plays a role in regulating long‐chain fatty acid metabolism in bovine mammary epithelial cells by modulating the expression of genes associated with fatty acid metabolic pathways.^[^ ^59^ ^]^ The report helps elucidate why genes associated with energy and lipid metabolism, such as those involved in “glycolysis/gluconeogenesis,” “pentose phosphate pathway,” and “lipid biosynthesis,” are expressed at higher levels in donkey sebocytes compared to those in humans, mice, and pigs. In addition, this may account for the higher abundance of carbohydrate and lipid metabolites observed in donkey skin. Thus, although we have identified a distinctive metabolic pattern in donkey skin, the precise relationship between these characteristic metabolites and the medicinal value of donkey‐hide gelatin have not yet been elucidated; this therefore requires further in‐depth study.

In summary, we have constructed a comprehensive multi‐tissue single‐cell transcriptome atlas for the adult Dezhou donkey and delineated specific transcriptional regulatory networks across various cells, tissues, and organ systems. Our study has elucidated the unique transcriptional patterns and metabolic characteristics of donkey sebocytes and highlighted the significant role that these cells have in the metabolic environment of donkey skin. This research not only provides valuable resources for the protection and utilization of donkey germplasm, but also addresses a critical gap in understanding the metabolic characteristics of donkey skin.

## 4 Experimental Section

### Ethical Statement

The management of experimental animals complied with the standard procedures outlined in the Guide for the Care and Use of Laboratory Animals (2010) and the principles of Animal Welfare Management (Public Law 99‐198). All animal experiments were approved by the Animal Ethics Committee of Qingdao Agricultural University (Approval No. 2021‐011).

### Collection of Animal Tissues

The experimental animals consisted of one female and two male Dezhou donkeys (Wutou strain), approximately two years old, provided by the national Dezhou donkey original breeding farm in Binzhou. The animals were bled and slaughtered using conventional humane methods after a 12 h fasting period. The isolated tissues were rinsed with 1× phosphate buffered saline (PBS) to remove impurities, immediately placed on ice, and transported to a sterile environment for subsequent experiments. Tissues were collected from 20 different organs, including the cerebrum, cerebellum, pineal gland, pituitary gland, heart, PBMC, lung, kidney, spleen, liver, pancreas, stomach, large intestine, small intestine, muscle, skin, testis, epididymis, ovary, and uterus.

### Single Cell/Nucleus Suspension Preparation and Sequencing

Fresh tissues were minced into small pieces and subsequently incubated in an enzymatic solution comprising 0.25% trypsin (Hyclone, Beijing, China) and collagenase (2 mg mL^−1^, Sigma‐Aldrich, C5138, Shanghai, China), maintained at a constant temperature of 37 °C for a duration of 15 min. Subsequently, 10% fetal bovine serum (FBS) was added to halt tissue digestion, and the samples were filtered through a 100 µm cell strainer. Subsequently, cells were resuspended in PBS containing 0.04% bovine serum albumin (BSA) and centrifuged at 500*g* for 5 min at 4 °C. This step was repeated three times to obtain a high‐quality cell suspension.

For the preparation of a single‐nuclear suspension, the tissue was first thawed, minced, and transferred to a Dounce homogenizer (TIANDZ) containing 1 mL of homogenization buffer composed of 250 × 10^−3^ m sucrose (Ambion), 10 mg mL^−1^ BSA (Ambion), 5 × 10^−3^ m MgCl_2_ (Ambion), 0.12  U µL^−1^ RNasin Plus (Promega, N2115), and 1× cOmplete Protease Inhibitor Cocktail (Roche, 11697498001). The tissue was then homogenized by gently tapping 25–50 times with a loose pestle. Subsequently, the mixture was filtered through a 40 µm cell sieve into a centrifuge tube and centrifuged at 500*g* for 5 min at 4 °C to pellet the nuclei. The pellet was then resuspended in buffer (320 × 10^−3^ m sucrose, 10 mg mL^−1^ BSA, 3 × 10^−3^ m CaCl_2_, 2 × 10^−3^ m magnesium acetate, 0.1 × 10^−3^ m ethylenediaminetetraacetic acid (EDTA), 10 × 10^−3^ m Tris‐HCl, 1 × 10^−3^ m dithiothreitol (DTT), 1× cOmplete Protease Inhibitor Cocktail, and 0.12 U µL^−1^ RNasein) to prepare a single‐nuclear suspension of 1000 nuclei µL^−1^.^[^ ^60^ ^]^

After obtaining single‐cell and single‐nucleus suspensions, a DNBelab C Series High‐throughput Single‐cell RNA Library Preparation Set V2.0 was used to process the suspensions for droplet generation, bead collection, reverse transcription, cDNA amplification, and purification to generate barcoded libraries. The DNBelab C library contains two components: the cDNA library and the oligo library, derived from mRNA capture magnetic beads and droplet recognition microbeads, respectively. After construction, the two libraries were sequenced simultaneously using the DNBSEQ T7 sequencing platform, employing the PE100 sequencing strategy.

### Quality Control and Preprocessing of Data with scRNA‐seq and snRNA‐seq

The donkey reference genome (Equus_asinus.ASM1607732v2.dna.toplevel.fa) and genome annotation file (Equus_asinus.ASM1607732v2.109.gtf) were downloaded from the ENSEMBL assembly. The reference genome index was then established using the “DNBC4tools mkref” function. Raw data were preprocessed using DNBC4tools (v2.1.0) to obtain single‐cell gene expression matrix and barcode information. Next, the single‐cell gene expression matrix was analyzed using Seurat software (v4.3.0). Environmental RNA contamination was removed using SoupX (v1.6.2; https://github.com/constantAmateur/SoupX), with all parameters set to default.^[^ ^61^ ^]^ In addition, doublet removal was performed using the default parameters of DoubletFinder (v2.0.3).^[^ ^62^ ^]^ In addition, cells with fewer than 200 genes and more than 30% mitochondrial genes were removed, and genes expressed in fewer than three cells were filtered out. High‐quality datasets were then generated.

Subsequently, data preprocessing was conducted using Seurat. In the standard pre‐processing workflow, data normalization was performed using the “NormalizeData” function, and highly variable genes were identified with the “FindVariableFeatures” function, the parameter was set with selection.method = “vst” and nfeatures = 2000. Next, batch correction was performed using the “FindIntegrationAnchors” function, and data integration was performed using the “IntegrateData” function.^[^ ^60^ ^]^ To achieve superior cell clustering results, the clustering results across various values of resolution parameters are evaluated using UMAP visualization and marker gene expression analysis. Based on these assessments, the “FindClusters” function with resolution parameter ranging from 0.8 to 1.5 was ultimately applied to delineate the cell clusters. Finally, visualization was performed using the “RunTSNE” and “RunUMAP” functions.

### Identification of Differentially Expressed Genes across Cell Clusters

Differential analysis of genes in cell clusters was performed using the “FindAllMarkers” function. Differentially expressed genes were required to meet the criteria “*p*‐value < 0.05; |Log_2_FC| > 0.25”.

### Cell Type Annotation

To finely define cell types of each tissue, every dataset from each tissue was analyzed independently. Specifically, the identity of each cluster was determined by combining differentially expressed gene sets identified through cell cluster differential analysis with typical cell type markers. The “FindAllMarkers” function was used to identify unbiased marker genes specific to each cell cluster, and GO enrichment analysis was performed with these marker genes, allowing to confirm cell type annotation through associated GO terms.

### Cell Cycle Index Estimation

The cell cycle status analysis utilized high confidence cell cycle markers compiled by Stephen et al., in combination with AUCell software (v1.22.0) to score the cycling and noncycling gene sets of each cell type.^[^ ^63^ ^]^ Finally, the cycling index was calculated based on the log ratio of the cycling number to noncycling cells for each cell type.

### Transcriptional Similarity Analysis between Cell Types

MetaNeighbor software (v1.20.0) was used to systematically evaluate transcriptional similarity among cell types both within and across species. Detailed scripts and tutorials for this analysis are accessible via the MetaNeighbor GitHub repository (http://github.com/gillislab/MetaNeighbor). Briefly, the single‐cell transcriptome data were firstly converted into SingleCellExperiment format, and a gene expression matrix was constructed, with rows representing alleles and columns representing cells. Highly variable genes were identified using “variableGenes” function in MetaNeighbor, and these genes were used as input for “MetaNeighbourUS” function, which was executed with the fast_version parameter set to TRUE. Subsequently, hierarchical clustering was performed based on the calculated AUROC scores (provided by MetaNeighbor and used as quantitative indexes of similarity between cell type pairs), to visually illustrate the relationships between cell types across and within species.^[^ ^20^ ^]^

### Cluster Analysis of Different Epithelial Cell Types

Differentially expressed genes between each type of epithelial cell were identified, and the average gene expression matrix for these gene sets in each cell type was calculated. Subsequently, the “dist” and “hclust” functions were used to calculate the distances between each cell type and complete the clustering.

### Gene Regulatory Network Inference

The donkey cisTarget database was created following the SCENIC standard process (https://github.com/aertslab/create_cisTarget_databases). The “tbl” file was constructed with reference to the JoGraesslin script (https://github.com/JoGraesslin/Zebrafish_SCENIC).

Gene regulatory networks specific to donkey organ systems, tissues, and cell types were constructed using pySCENIC.^[^ ^24^ ^]^ Specifically, the gene regulatory network was firstly inferred using the GRBboost2 algorithm, and cis‐regulatory motif analysis was performed on each co‐expression module using the “pyscenic ctx” function. Subsequently, target genes of the TFs were scored using AUCell. Finally, TFs specific to each donkey organ system, tissue, and cell type were identified using the “calcRSS” function. TFs were considered significant when their AUCell score was >0.1 and Z‐score normalized RSSZ > 1.0.^[^ ^25^ ^]^

Tissue‐specific TF modules were identified following the method proposed by Suo et al.^[^ ^25^ ^]^ First, the Pearson correlation coefficient (PCC) of each pair of TFs was computed based on TF activity scores of each cell. For a given pair of regulons A and B, the corresponding CSI was defined as the fraction of regulons whose PCC with both A and B were lower than the PCC between A and B. Finally, TF modules were constructed using Ward clustering on data matrices with CSI > 0.9.

### High‐Dimensional Weighted Gene Co‐Expression Network Analysis

The hdWGCNA was used to identify characteristic gene modules of organ systems. The process was as follows: First, genes expressed in at least 5% of cells were selected to create WGCNA objects, and “metacells” were constructed using the “MetacellsByGroups” function to reduce sparsity while retaining cell heterogeneity. Next, the optimal soft power threshold for constructing the co‐expression network was calculated using the “TestSoftPowers” function. In this study, a soft threshold of 7 was determined to best fit the network structure. Subsequently, the “ModuleEigengenes” function was utilized to calculate module eigengenes. Finally, UMAP was used to embed the co‐expression network topological overlap matrix (TOM) into a two‐dimensional manifold.^[^ ^21^ ^]^

### Cross‐Species Comparison of Multi‐Tissue Cell Landscapes

For the comparative analysis of multi‐tissue scRNA‐seq data from donkeys, humans, pigs, and mice, high‐quality data from nine tissues in these four species, including the cerebrum, cerebellum, heart, kidney, liver, lung, PBMC, skin, and spleen, were first selected. Human, pig, and mouse data were downloaded from public databases: human cerebrum, GSE97930;^[^ ^64^ ^]^ human cerebellum, heart, kidney, lung, and PBMC, GSE134355;^[^ ^65^ ^]^ human liver, GSE136103;^[^ ^66^ ^]^ human skin, GSE173252;^[^ ^67^ ^]^ human spleen, GSE159929;^[^ ^68^ ^]^ pig cerebrum, cerebellum, heart, kidney, liver, lung, PBMC, and spleen: GSE193975;^[^ ^69^ ^]^ pig skin: GSE225416;^[^ ^70^ ^]^ mouse cerebrum, heart, kidney, liver, lung, and spleen, GSE176063;^[^ ^71^ ^]^ mouse cerebellum, CNP0000892;^[^ ^72^ ^]^ mouse PBMC, GSE108097;^[^ ^73^ ^]^ and mouse skin, GSE185268.^[^ ^74^ ^]^ Subsequently, all data were processed using the pipeline in the “Quality control and preprocessing of scRNA‐seq and snRNA‐seq” section. Upon securing high‐quality data, the Ensembl Biomart tool was used to convert the gene names of donkeys, pigs, and mice into their human orthologous genes. The reciprocal principal component analysis (RPCA) method was then used to integrate the cross‐species scRNA‐seq data of the four species.

To compare the cell type similarity between donkeys and the other three species, the average expression values of the top 2000 highly variable genes in each cell type across the different species were calculated, and correlations were determined using Spearman's correlation coefficient.

### Evaluation of Metabolic Activity

Activity scoring of metabolic pathways in different epithelial cell types was performed following the pipeline Single‐Cell‐Metabolic‐Landscape (https://github.com/LocasaleLab/Single‐Cell‐Metabolic‐Landscape). First, the mean expression level of each metabolic gene in each cell type was calculated. Then, the expression level of each gene in that cell type was compared to the mean expression level of that gene across all cell types to obtain the relative expression level. For each metabolic pathway, the weighted average expression level of the genes included in the pathway was calculated. To ensure that pathway activity was not affected by genes with low expression levels or high deletion rates, only genes with nonzero expression levels and expressed in at least 25% of the cells were used. Finally, a random permutation test was conducted to assess the statistical significance of pathway activity in a specific cell type.^[^ ^34^ ^]^

In addition, scFEA was used to infer the cell‐wise fluxome from scRNA‐seq data. After obtaining the epithelial cell count matrix, the donkey gene names were converted to human orthologous genes. Subsequently, the metabolic fluxome and abundance of each type of epithelial cell were calculated using “scFEA.py.”^[^ ^35^ ^]^

### GO and KEGG Enrichment Analysis

GO and KEGG enrichment analysis of specific gene sets were performed using g:Profiler (https://biit.cs.ut.ee/gprofiler).^[^ ^75^ ^]^ Terms with a *p*‐value < 0.05 were considered statistically significant.

### Metabolome Detection of Skin

Skin samples were collected from the area above the last rib of five adult Dezhou donkeys (Wutou strain), Mongolian horses, Bohai black cattle, and pigs (three‐way hybrid of Landrace, Large White, and Duroc). The samples were collected from the same anatomical location as those used for the single‐cell transcriptome analysis of donkey skin. Donkey and horse skin samples were sourced from Dezhou Demu Meat Processing Co., Ltd. Cattle skin samples were obtained from Yangxin Niuzhigu Industrial Park, while pig skin samples were provided by Qingdao Wanfu Pig Breeding Base. Fresh skin samples were shaved to remove hair and subcutaneous fat before being immediately stored in liquid nitrogen. The LC/MS system for metabolomics analysis was composed of a Waters Acquity I‐Class PLUS ultra‐high‐performance liquid tandem and a Waters Xevo G2‐XS QT of high resolution mass spectrometer. The column used was a Waters ACQUITY UPLC BEH Amide column (1.7 µm, 2.1 mm × 100 mm). Subsequently, the metabolite levels for each species were normalized to determine the highest and lowest values. These values were then divided into tertiles, categorizing the levels as high, intermediate, and low, respectively.

Differential metabolites based on the grouping information were calculated and compared. First, the *p*‐value for the significance of each compound was calculated using the *t*‐test. Then, the variable importance in the projection (VIP) value of the model was determined through multiple cross‐validation. Differential metabolites were required to meet the criteria of “*p*‐value <0.05, VIP >1”. Finally, MetaboAnalyst (v6.0) and Reactome were utilized to perform enrichment analysis on the differential metabolites.^[^ ^36^, ^76^ ^]^

PCA of the metabolome was performed using FactoMineR software (v2.11) with default parameters. Hierarchical clustering analysis was conducted using the “dist” function with the “euclidean” parameter to calculate the distance matrix, followed by the “hclust” function with the “average” parameter to perform hierarchical clustering.

### Weighted Correlation Network Analysis

The co‐expression network was constructed using 372 metabolites with the WGCNA R package (v1.72). Metabolite modules were defined using the Dynamic Tree Cut (dynamicTreeCut R package; v1.63). Associations between module eigengenes and species were evaluated by Wilcoxon rank sum. Key metabolites within the modules were identified using module membership and metabolite significance, with thresholds set at “module membership” >0.9 and “metabolite significance” >0.9.^[^ ^37^ ^]^

### Cell–Cell Interaction Analysis

The interaction network among cell populations in donkey skin was analyzed using CellChat software (v1.6.1). According to the protocol, the single‐cell gene count matrix generated by Seurat served as the input for CellChat.^[^ ^40^ ^]^ The “computeCommunProb” function was used to infer the cell–cell communication network for each ligand‐receptor pair and signaling pathway. Subsequently, the “aggregateNet” function was utilized to calculate the frequency and strength of cell‐cell interactions between different cell types.

To investigate the effects of *CD44* within sebocytes, NicheNet (v2.0.5) analysis was initially conducted to simulate the intercellular communication network between sebocytes and other skin cell types in donkey skin.^[^ ^41a^ ^]^ This analysis specifically focused on identifying target genes of *CD44* associated with intercellular signaling in sebocytes. The methodology adhered to guidelines publicly provided at NicheNet GitHub vignette (https://github.com/saeyslab/nichenetr/tree/master/vignettes). Sebocytes were designated as receptor cells, while endothelial cells, fibroblasts, keratinocytes, macrophages, mast cells, melanocytes, pericytes, smooth muscle cells, and sweat gland epithelial cells were defined as sender cells. Using the normalized expression data of the identified gene set, a regulatory network analysis was subsequently performed with GENIE3 (v1.28.0) to predict target genes regulated by *CD44* in sebocytes.^[^ ^41b^ ^]^

### Hematoxylin and Eosin (H&E) Staining and Immunostaining

Tissues were placed in 4% paraformaldehyde at 4 °C overnight. Dehydration and paraffin embedding were performed the next day and 5‐µm‐thick sections were cut. For morphological analysis, sections were stained with hematoxylin and eosin. For IF, sections were treated with sodium citrate at 96 °C for 10 min, followed by blocking with TBST containing 5% BSA for 30 min at room temperature (RT). After blocking, sections were incubated with the primary antibody overnight at 4 °C. The following day, sections were incubated with secondary antibodies for 1 h at 37 °C (Table S11, Supporting Information). Finally, DAPI was used for nuclear staining. For IHC, the same procedure was followed until the blocking step, then sections were treated with 3% H_2_O_2_ for 10 min. Subsequently, slides were incubated with the primary antibody overnight at 4 °C, followed by incubation with the secondary antibody for 1 h at RT (Table S11, Supporting Information), and finally stained with peroxidase substrate and counterstained with hematoxylin. Images were examined using an Olympus microscope (Olympus, BX51, Japan).

### Western Blot Analysis

Skin tissues from donkeys, horses, cattle, and pigs were placed in radioimmunoprecipitation assay (RIPA) lysis buffer (Beyotime, P0013B, Shanghai, China), and proteins were extracted using a tissue homogenizer (TIANGEN, OSE‐Y30, Beijing, China). The extracted proteins were then separated by SDS‐polyacrylamide gel electrophoresis (SDS‐PAGE) and transferred to polyvinylidene fluoride (PVDF) membranes (Millipore, ISEQ00010, USA). After transfer, the membranes were blocked at RT for 2 h, then incubated with primary antibodies at 4 °C overnight, and secondary antibodies at RT for 2 h (Table S11, Supporting Information). Finally, protein band signals were captured using a Tanon 5200 imaging system (Tanon, Shanghai, China), and grayscale values were calculated using AlphaView SA software (ProteinSimple, California, USA).

### Statistical Analysis

Statistical analyses were performed using GraphPad Prism 8 software. Results were delivered as mean ± standard deviation (SD) from five independent experiments. One‐way analysis of variance (ANOVA) was used to assess differences among groups, followed by Tukey's honest significant difference (Tukey's HSD) test as the post‐hoc test method for pairwise comparisons of group means.

## Conflict of Interest

The authors declare no conflict of interest.

## Author Contributions

Conceptualization: Y.T., J.W., and W.S.; Investigation: S.L., and J.W.; Methodology: Y.T., and J.W.; Resources: Y.T., J.L., H.L., N.C., Z.F., and W.S.; Supervision: S.L., Y.S., T.Z., J.W., and W.S.; Visualization: Y.T., H.S., and X.W.; Writing – original draft, Y.T.; Writing – review & editing: Y.T., J.W., T.Z., and W.S.

## Code Availability

All code used for the processing of single‐cell transcriptome and metabolome data has been made publicly available on GitHub and available through this URL: https://github.com/Donkey‐Cell‐Atlas/A‐reference‐single‐cell‐transcriptomic‐atlas‐of‐Dezhou‐donkey.

## Supporting information



Supporting Information

Supplemental Table 1

Supplemental Table 2

Supplemental Table 3

Supplemental Table 4

Supplemental Table 5

Supplemental Table 6

Supplemental Table 7

Supplemental Table 8

Supplemental Table 9

Supplemental Table 10

Supplemental Table 11

## Data Availability

The single‐cell and single‐nuclei RNA sequencing data generated in this study have been deposited in the Genome Sequence Archive (GSA, https://ngdc.cncb.ac.cn/gsa) under the accession code “CRA018235.” In addition, the skin metabolomics data have been deposited in the Open Archive for Miscellaneous Data (OMIX, https://ngdc.cncb.ac.cn/omix/) and can be accessed using the following accession codes: OMIX007259, OMIX007260, OMIX007261, and OMIX007262.
