# Supplementary material for: Revealing the Transcriptional and Metabolic Characteristics of Sebocytes Based on the Donkey Cell Transcriptome Atlas
Source: Adv Sci (Weinh). 2025 Feb 27;12(16):2413819. doi: 10.1002/advs.202413819 (PMC12021041; doi:10.1002/advs.202413819)
Supplement: Supplementary file 1 — Supporting Information [file ADVS-12-2413819-s007.pdf]

ADVANCED SCIENCE
Open Access

Supporting Information

for *Adv. Sci.*, DOI 10.1002/advs.202413819

Revealing the Transcriptional and Metabolic Characteristics of Sebocytes Based on the Donkey Cell Transcriptome Atlas

Yu Tian, Shuqin Liu, Hongtao Shi, Jianjun Li, Xinglong Wan, Yujiang Sun, Huayun Li, Ning Cao, Zhixi Feng, Teng Zhang, Junjie Wang* and Wei Shen**

Supporting Information

Revealing the transcriptional and metabolic characteristics of sebocytes based on the donkey cell transcriptome atlas

Yu Tian [1,2], Shuqin Liu [1], Hongtao Shi [3], Jianjun Li [4], Xinglong Wan [3], Yujiang Sun [1], Huayun Li [5], Ning Cao [5], Zhixi Feng [5], Teng Zhang [2,*], Junjie Wang [1,*], Wei Shen [1,*]

[1] College of Animal Science and Technology, Qingdao Agricultural University, Qingdao 266109, China;

[2] State Key Laboratory of Reproductive Regulation and Breeding of Grassland Livestock (R2BGL), College of Life Sciences, Inner Mongolia University, Hohhot 010070, China;

[3] School of Science and Information Science, Qingdao Agricultural University, Qingdao 266109, China;

[4] National Dezhou Donkey Original Breeding Farm, Binzhou 251903, China;

[5] Annoroad Gene Technology, Beijing 100176, China.

* Correspondence and reprint requests to: Prof. Wei Shen; E-mail: wshen@qau.edu.cn

Prof. Junjie Wang; E-mail: 202101017@qau.edu.cn

Prof. Teng Zhang; E-mail: zhangteng428@imu.edu.cn

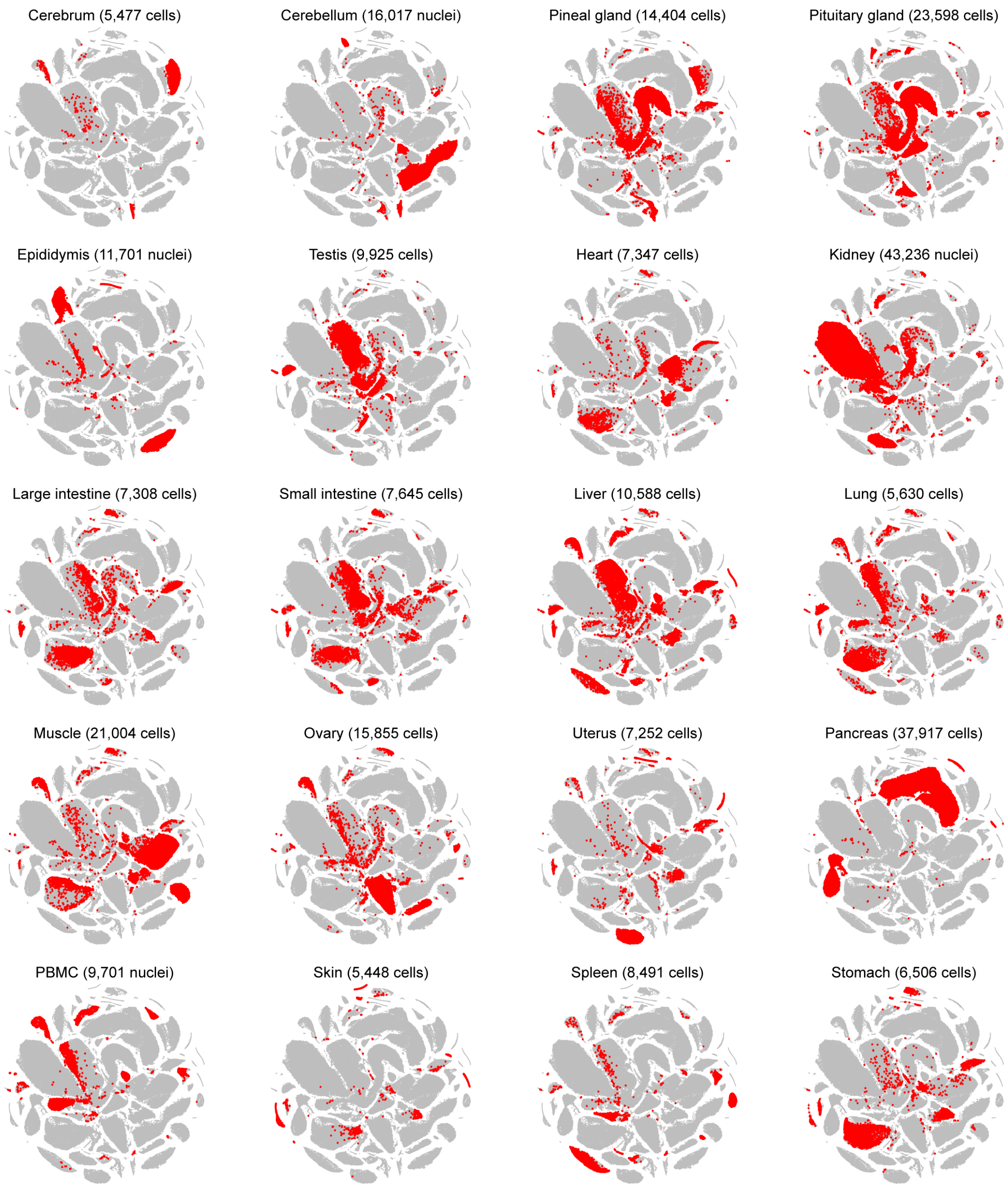

Figure S1

Global profiling of individual donkey tissues. tSNE projection of the global clustering indicating the distribution of all cells (highlighted in red) from individual tissues. The cell number profiled for each tissue is indicated in every panel.

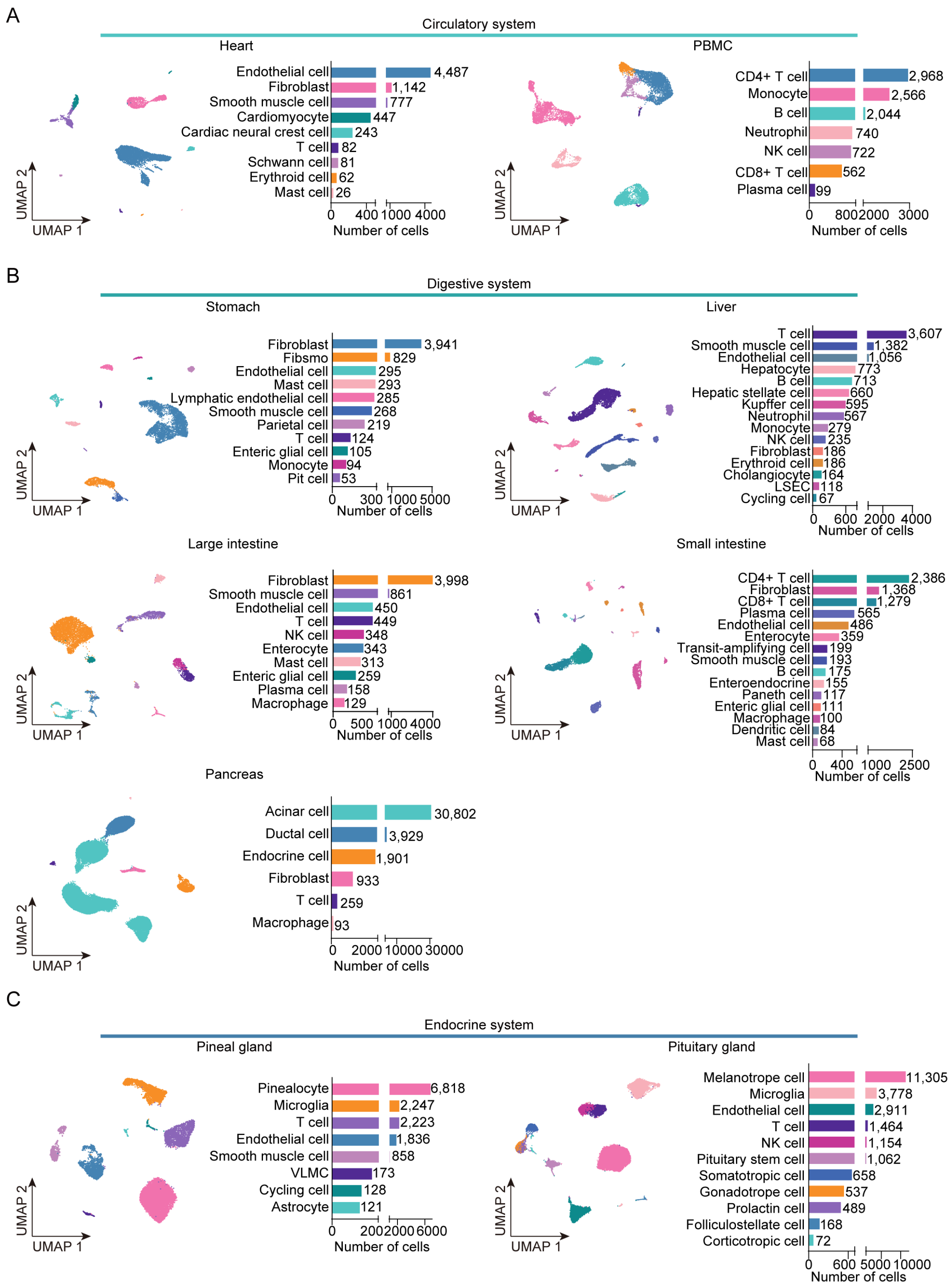

Figure S2

Visualization and annotation of the 84 major donkey cell types - 1. UMAP plots show cell clustering for each organ in the (A) circulatory system (heart and PBMC), (B) digestive system (stomach, liver, large intestine, small intestine, and pancreas), and (C) endocrine system (pineal

gland and pituitary gland) as well as quantification of each cell type within each organ.

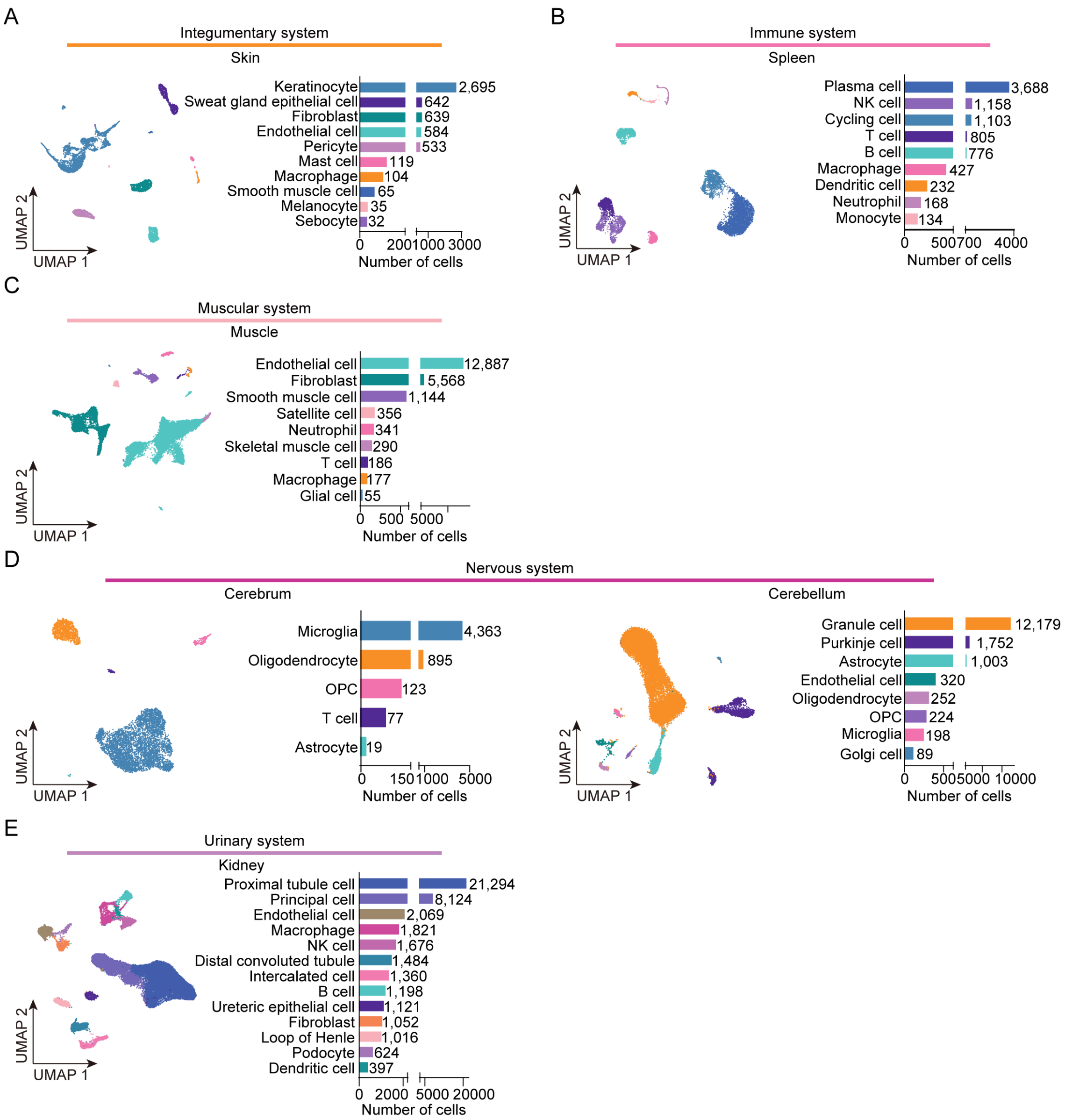

Figure S3

Visualization and annotation of the 84 major donkey cell types - 2. UMAP plots show cell clustering for each organ in the (A) integumentary system (skin), (B) immune system (spleen), (C) muscular system (muscle), (D) nervous system (cerebrum and cerebellum), and (E) urinary system (kidney) as well as quantification of each cell type within each organ.

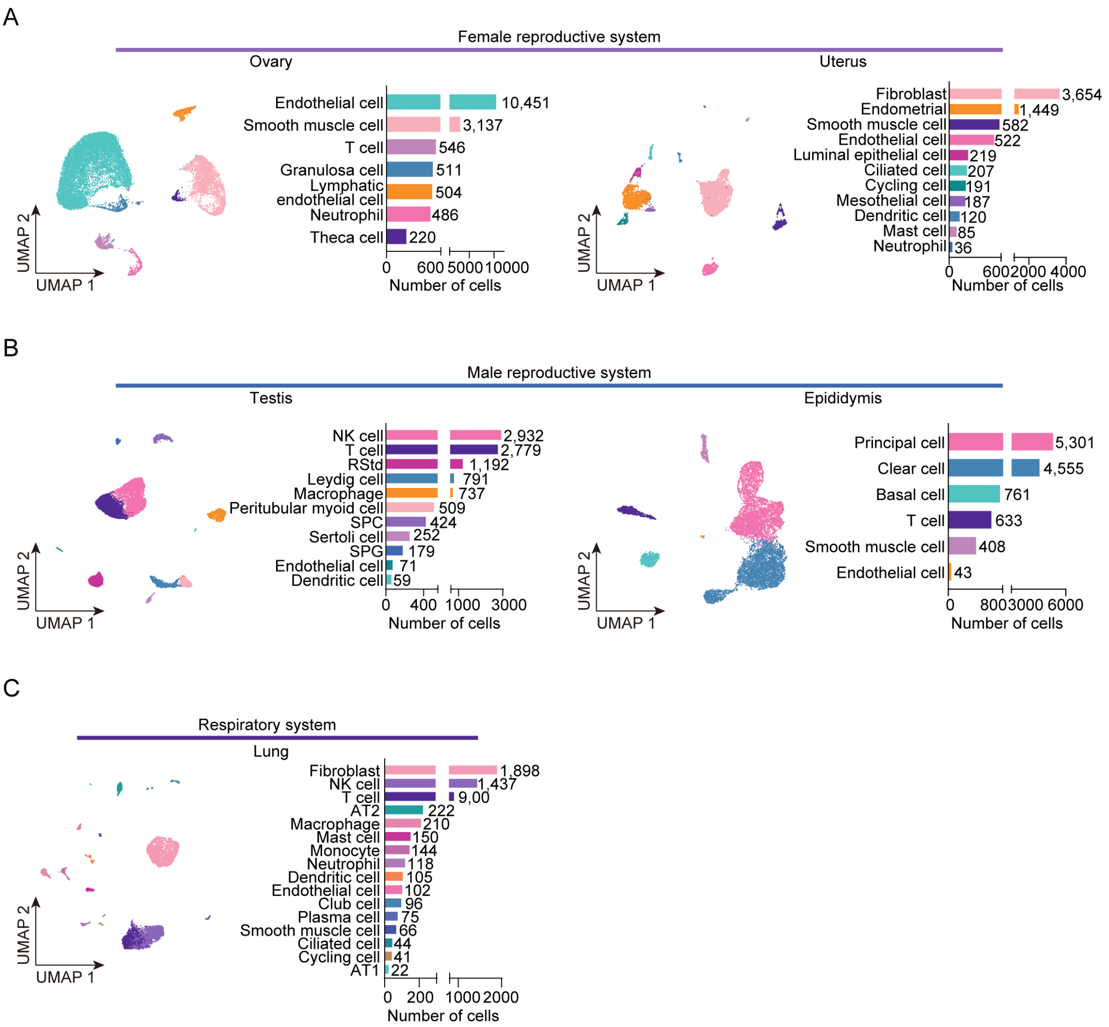

Figure S4

Visualization and annotation of the 84 major donkey cell types - 3. UMAP plots show cell clustering for each organ in the (A) female reproductive system (ovary and uterus), (B) male reproductive system (testis and epididymis), and (C) respiratory system (lung) as well as quantification of each cell type within each organ.

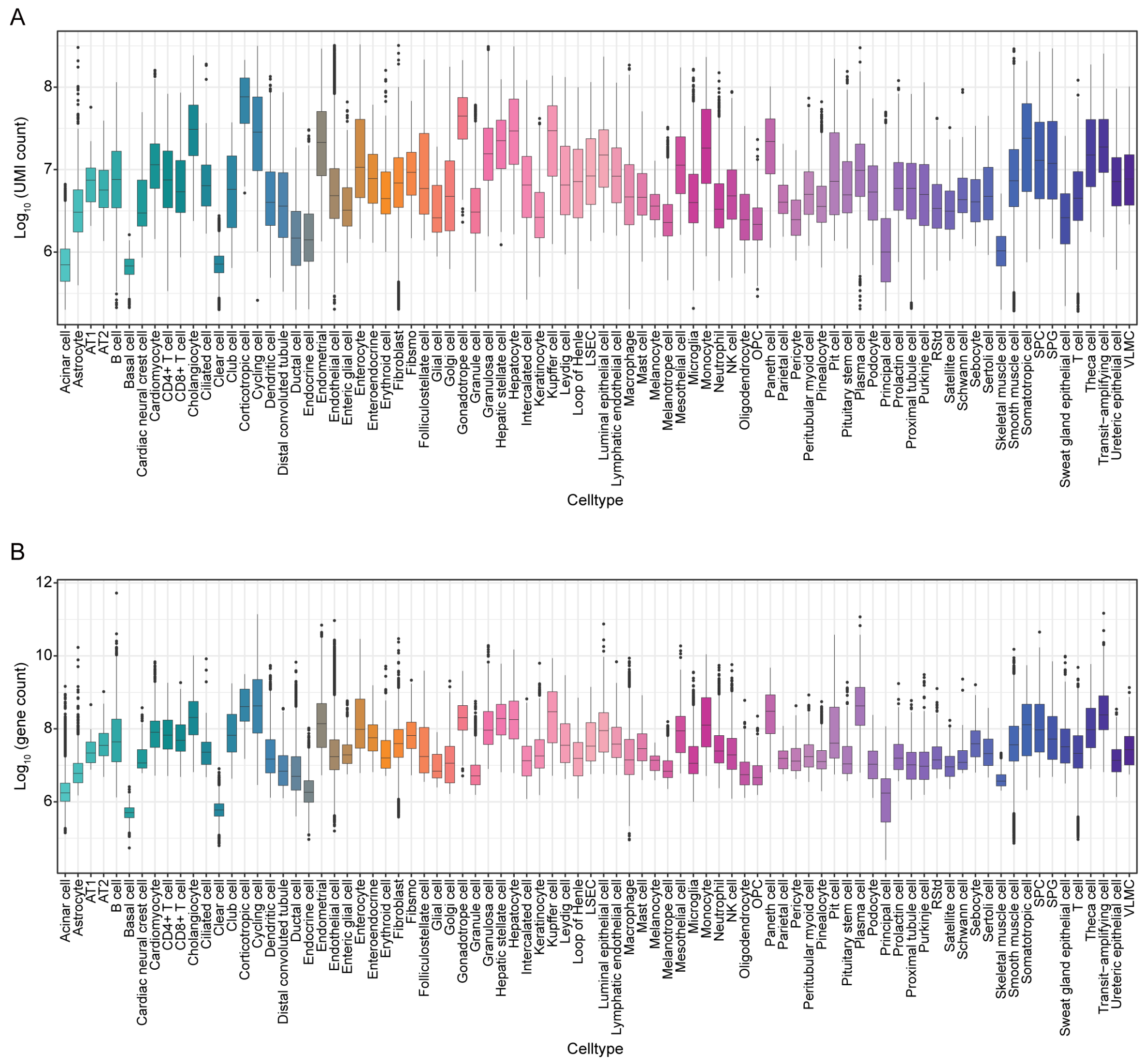

Figure S5

UMI and gene count of the donkey cell types. A) Boxplot showing the number of UMI detected in each major annotated cell type. B) Boxplot showing the number of gene detected in each major annotated cell type.

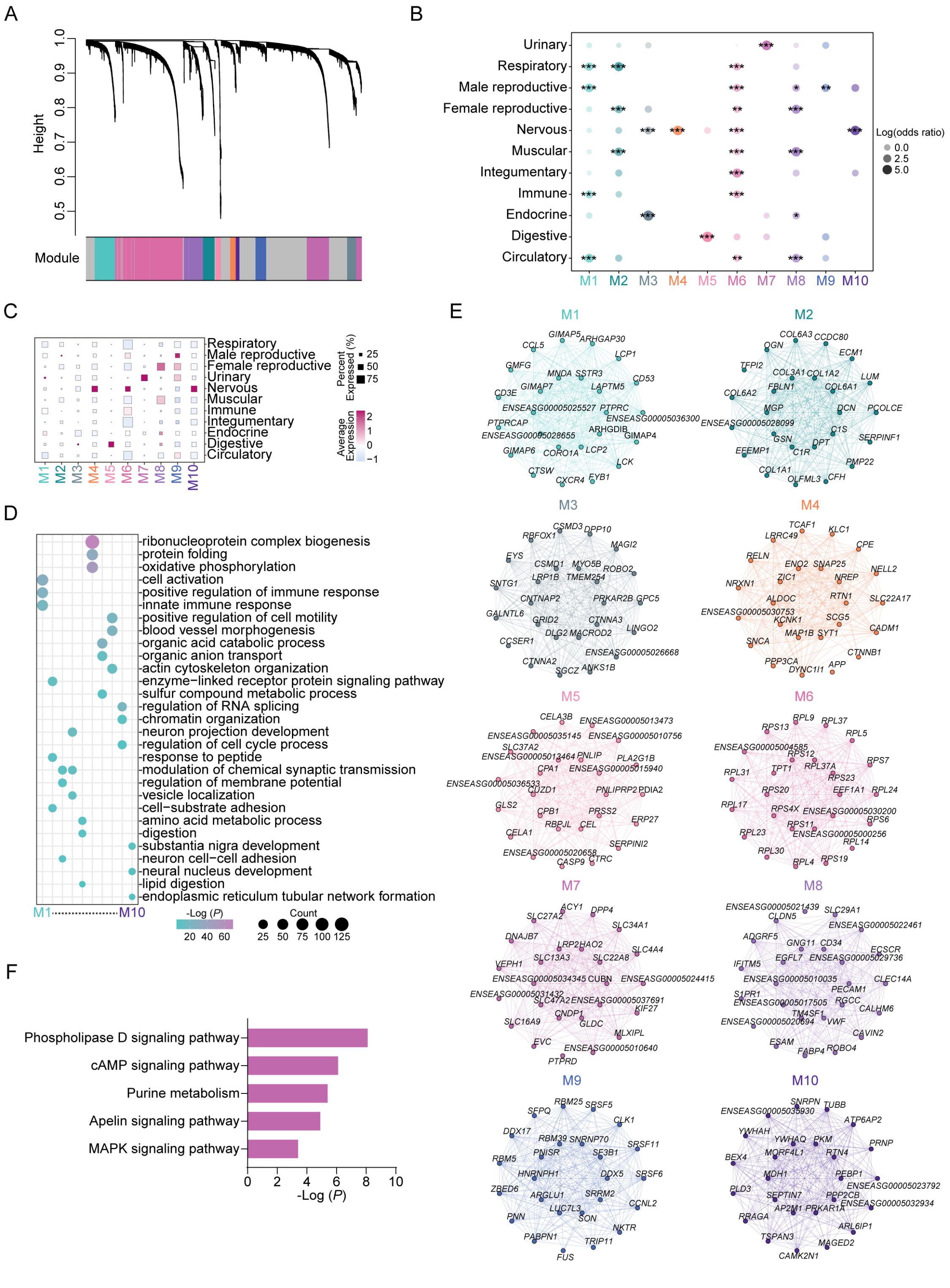

Figure S6

Co-expression network analysis of donkey system. A) Visualize the 10 modules in a scale-free network using a dendrogram. B) Co-expression modules were compared with marker genes of donkey organ system. The size of the dots represents the number of overlapping genes. FDR

significance levels as stars on top of the dots. “***”: 0 - 0.001; “**”: 0.001 - 0.01; “*”: 0.01 - 0.05; (No symbol): > 0.05. C) Heatmap showing expression levels of co-expression modules in different organ system. D) Co-expression plots for 10 modules. E) Dot plot showing the GO enrichment results for each co-expression module. F) KEGG enrichment analysis results of gene sets within module 3.

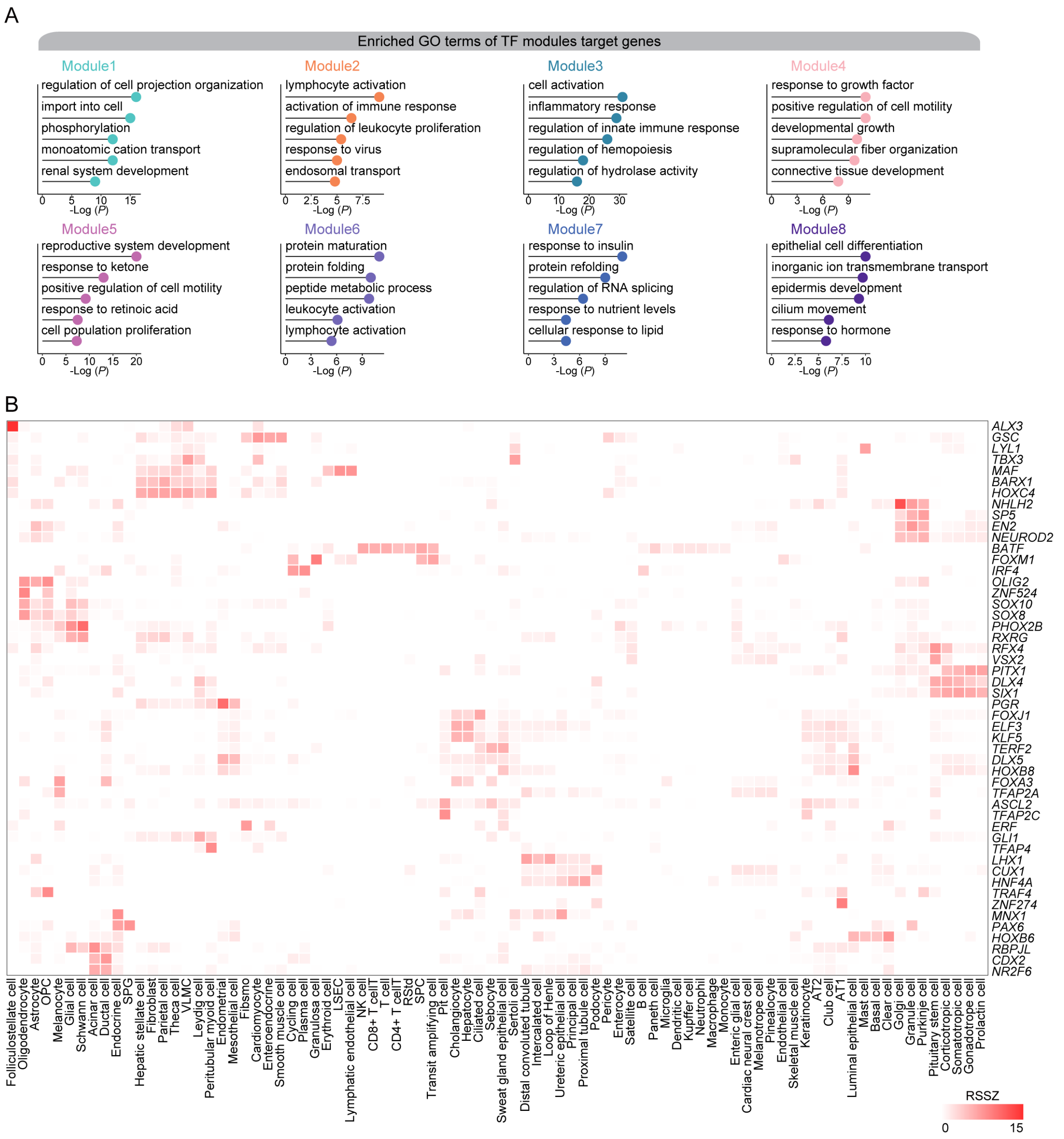

Figure S7

Cell-type-specific transcriptional gene regulatory network. A) GO analysis of target genes of TF modules. B) Heatmap showing TFs enriched in different cell categories. Color depth represents the level of RSSZ.

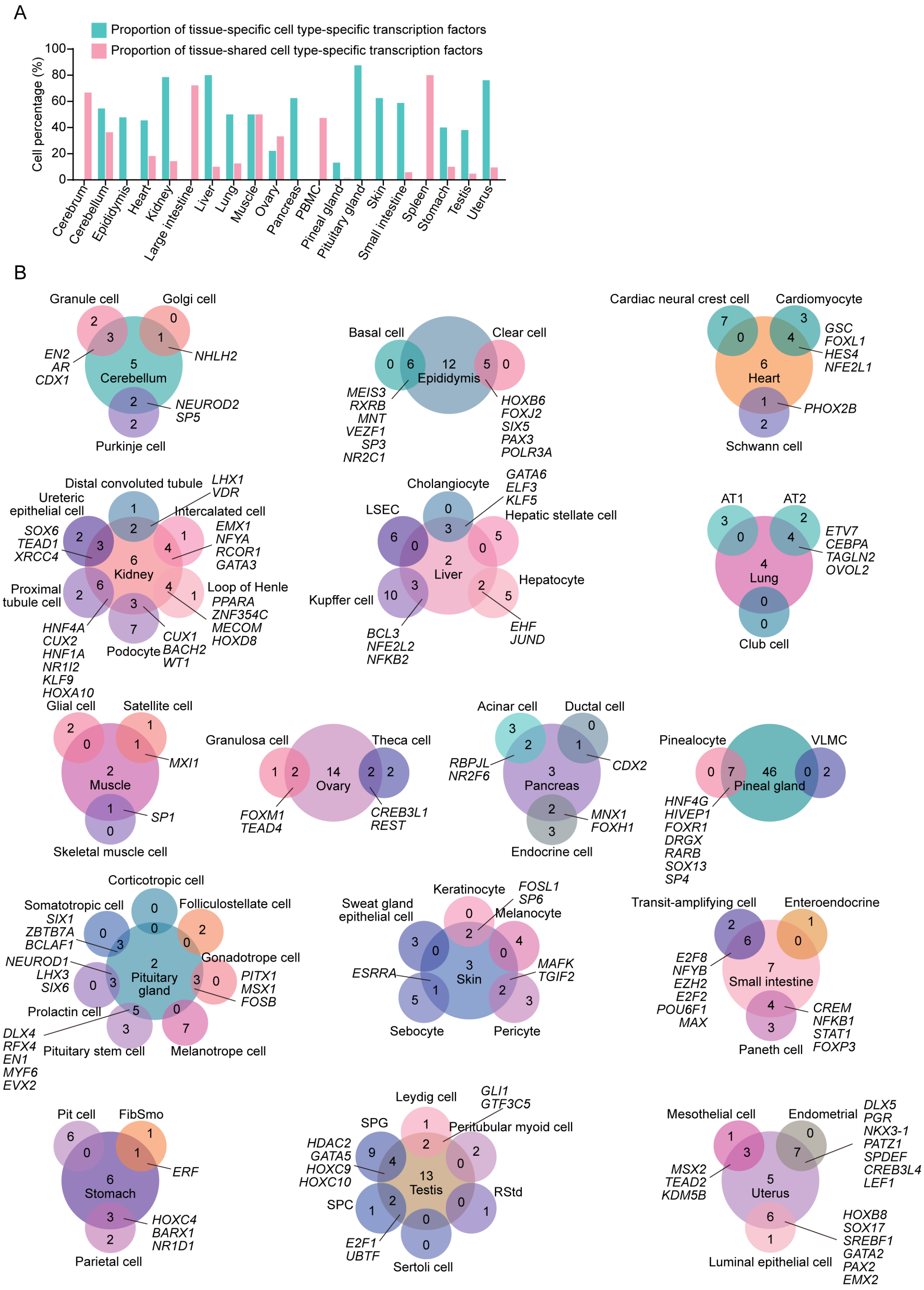

Figure S8

Comparison between tissue-specific and cell-type-specific transcription factors. A) The

proportion of tissue-specific cell type-specific transcription factors (CSTFs) compared to tissue-shared CSTFs. B) Venn diagram showing the overlap between tissue-specific TFs and tissue-specific CSTFs in each tissue.

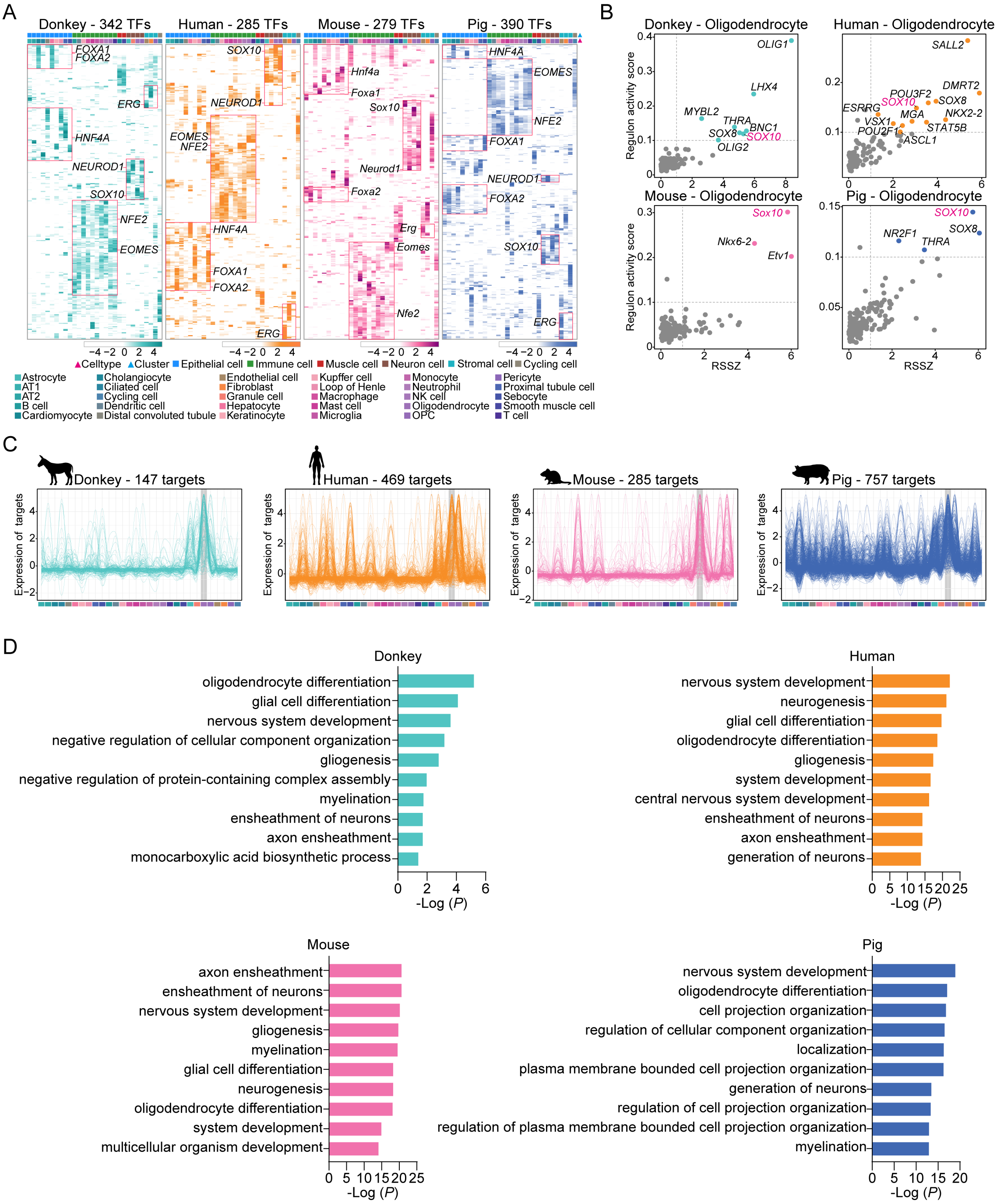

Figure S9

Conservation of cell types across species. A) Heatmap illustrating the transcription factor RSSZ across various cell types in the donkey, human, mouse, and pig. B) Plots showing regulon activity score and RSSZ in oligodendrocytes in the donkey, human, mouse, and pig. C) Graphs illustrating the expression patterns of *SOX10* target genes across four mammalian

species. Cell type coloring is consistent with (A). D) Biological functions regulated by *SOX10* target genes in mammalian.

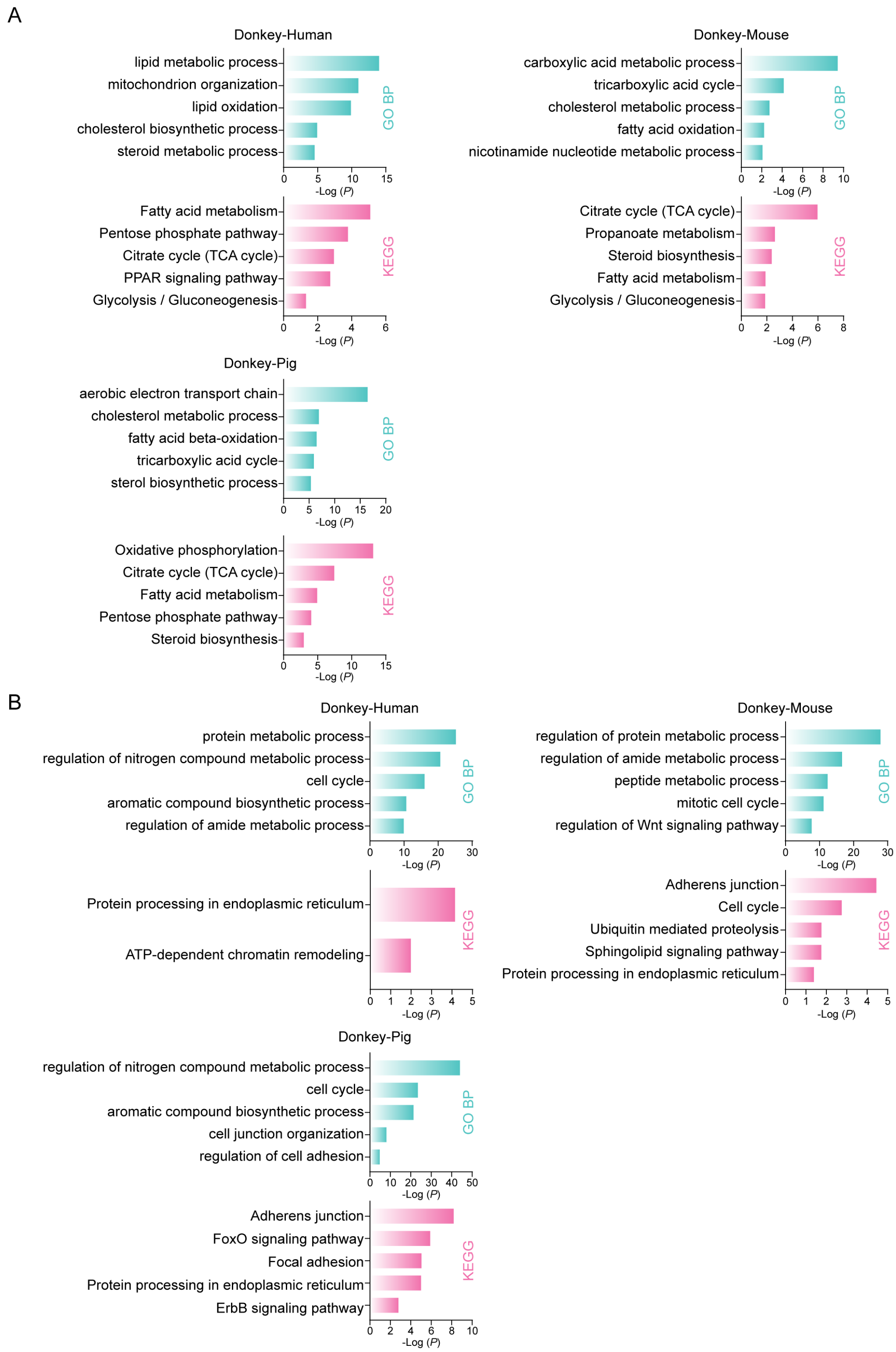

Figure S10

GO and KEGG enrichment analysis results of differentially expressed genes in sebocytes of donkeys and other species. A) GO and KEGG enrichment analysis results of genes with

significantly upregulated expression levels in donkey sebocytes. B) Results of GO and KEGG enrichment analysis of genes with significantly upregulated expression in human, mouse and pig sebocytes.

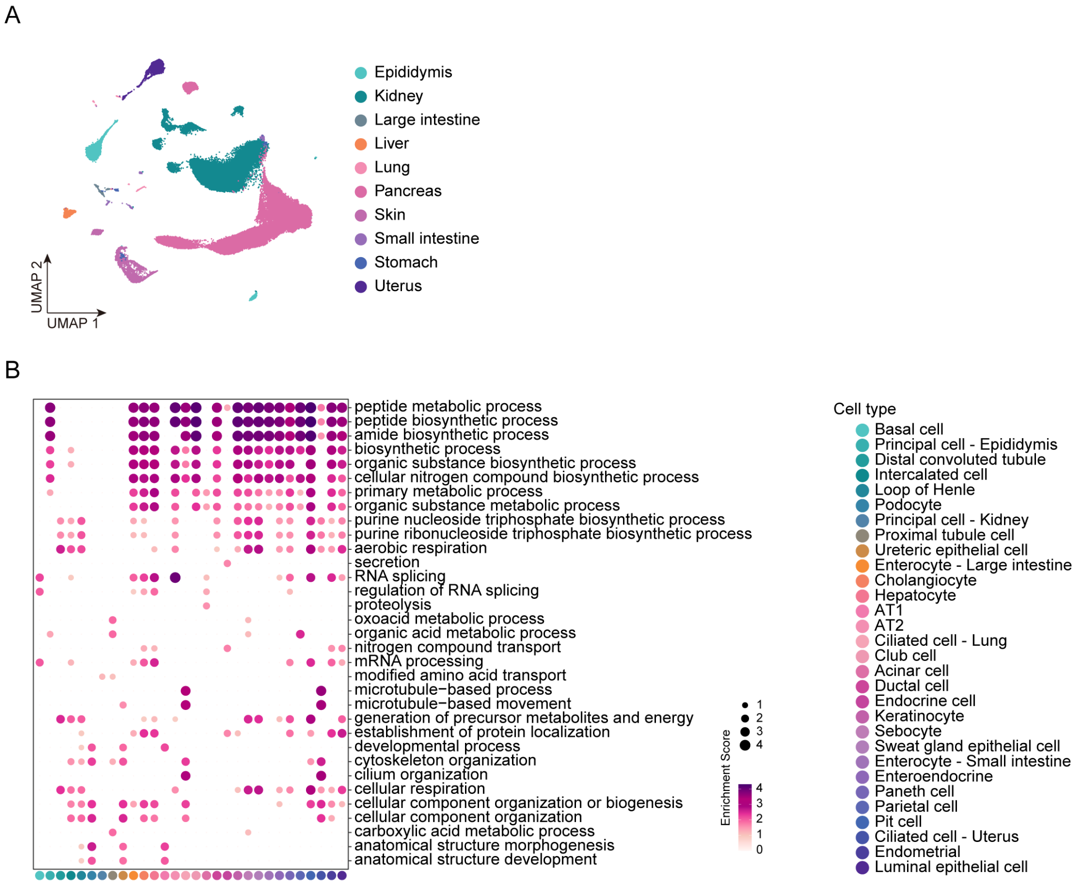

Figure S11

Comparative analysis of epithelial cells between tissues. A) UMAP plot showing clustering results of epithelial cells colored by tissue origin. B) GO enrichment analysis results of highly expressed genes specific to different epithelial cell types.

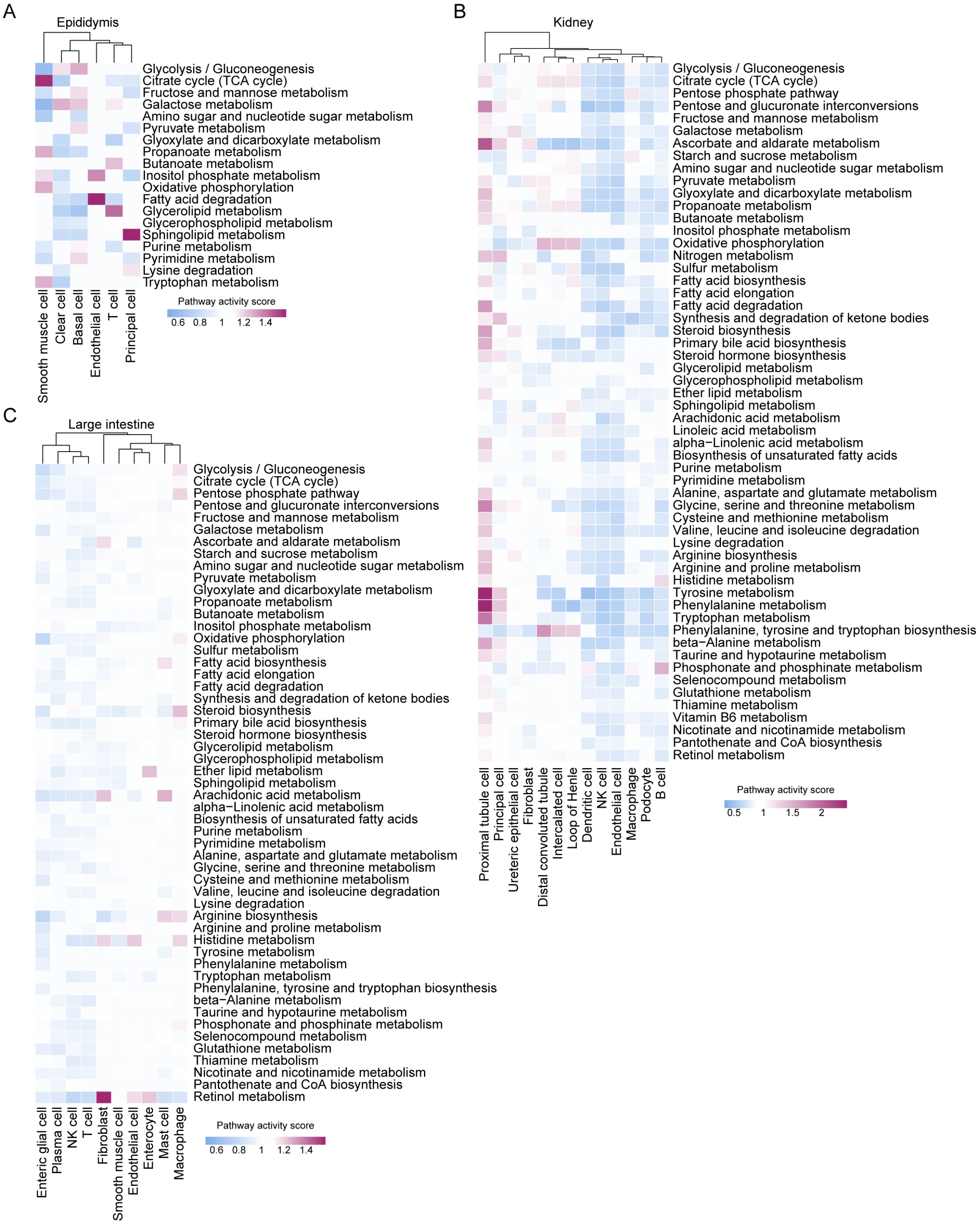

Figure S12

Cell type-specific metabolic features - 1. Heatmap showing metabolic pathway activity score for cell types in the (A) epididymis, (B) kidney, and (C) large intestine datasets.

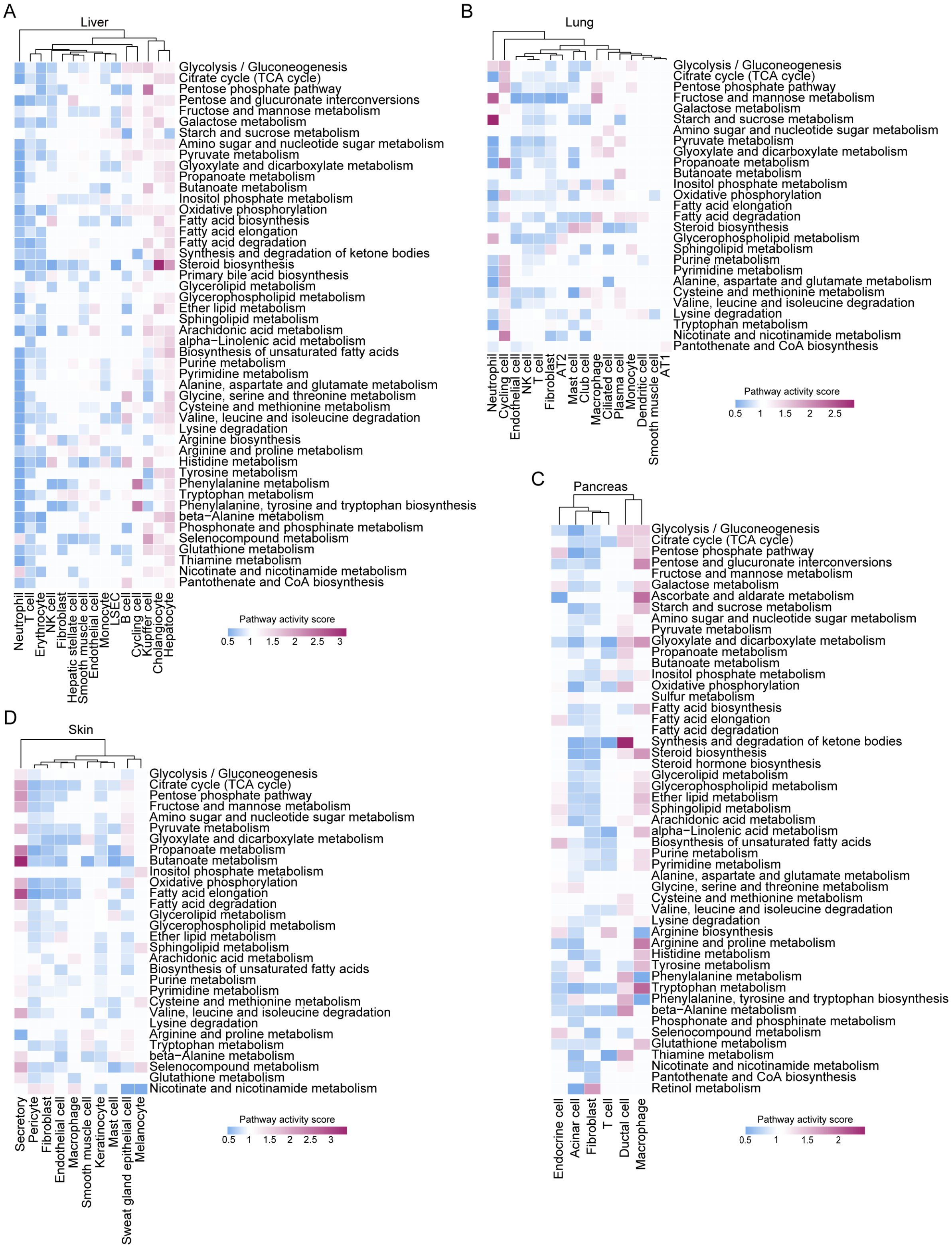

Figure S13

Cell type-specific metabolic features - 2. Heatmap showing metabolic pathway activity score for cell types in the (A) liver, (B) lung, (C) pancreas, and (D) skin datasets.

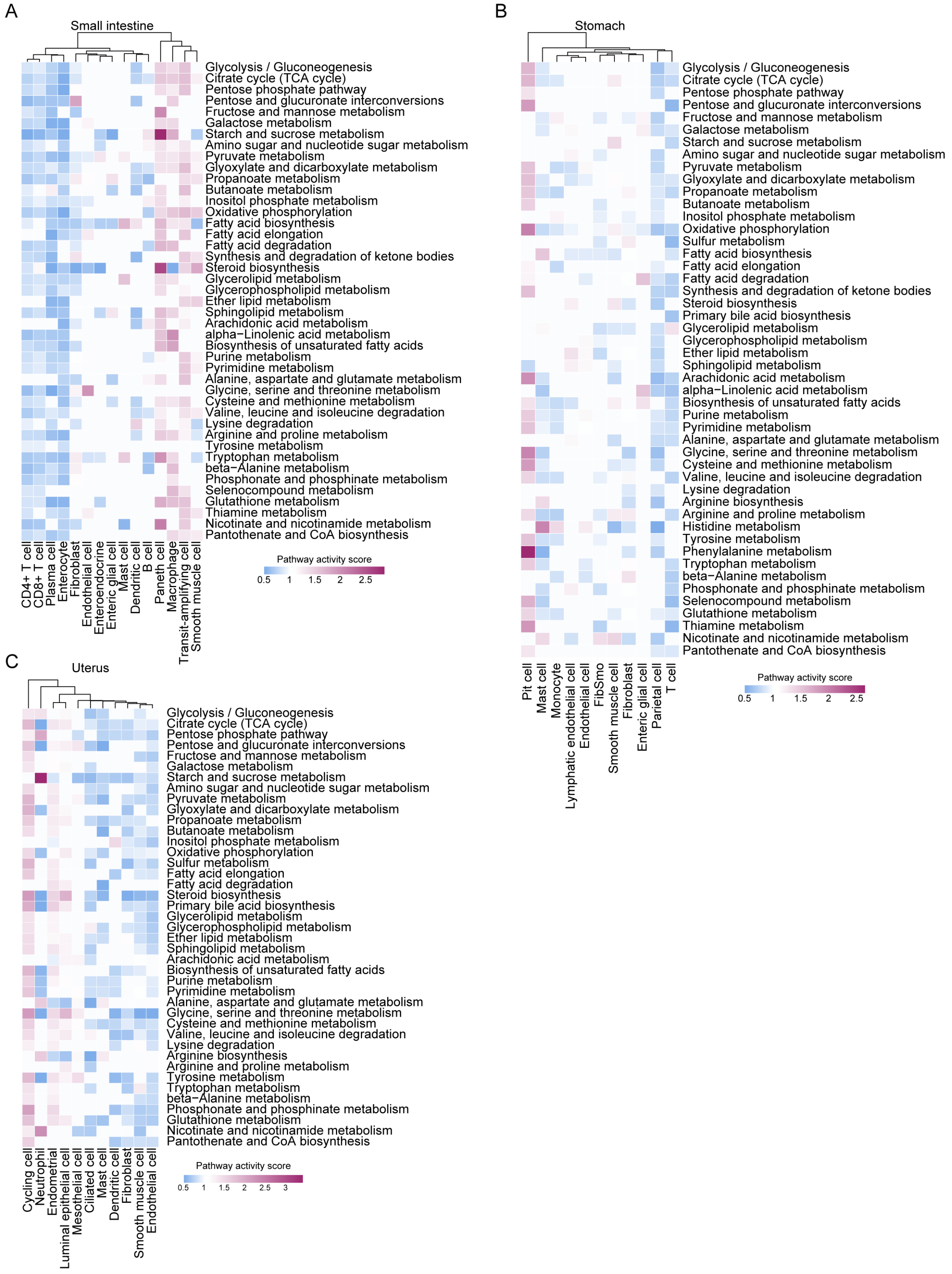

Figure S14

Cell type-specific metabolic features - 3. Heatmap showing metabolic pathway activity score

for cell types in the (A) small intestine, (B) stomach, and (C) uterus datasets.

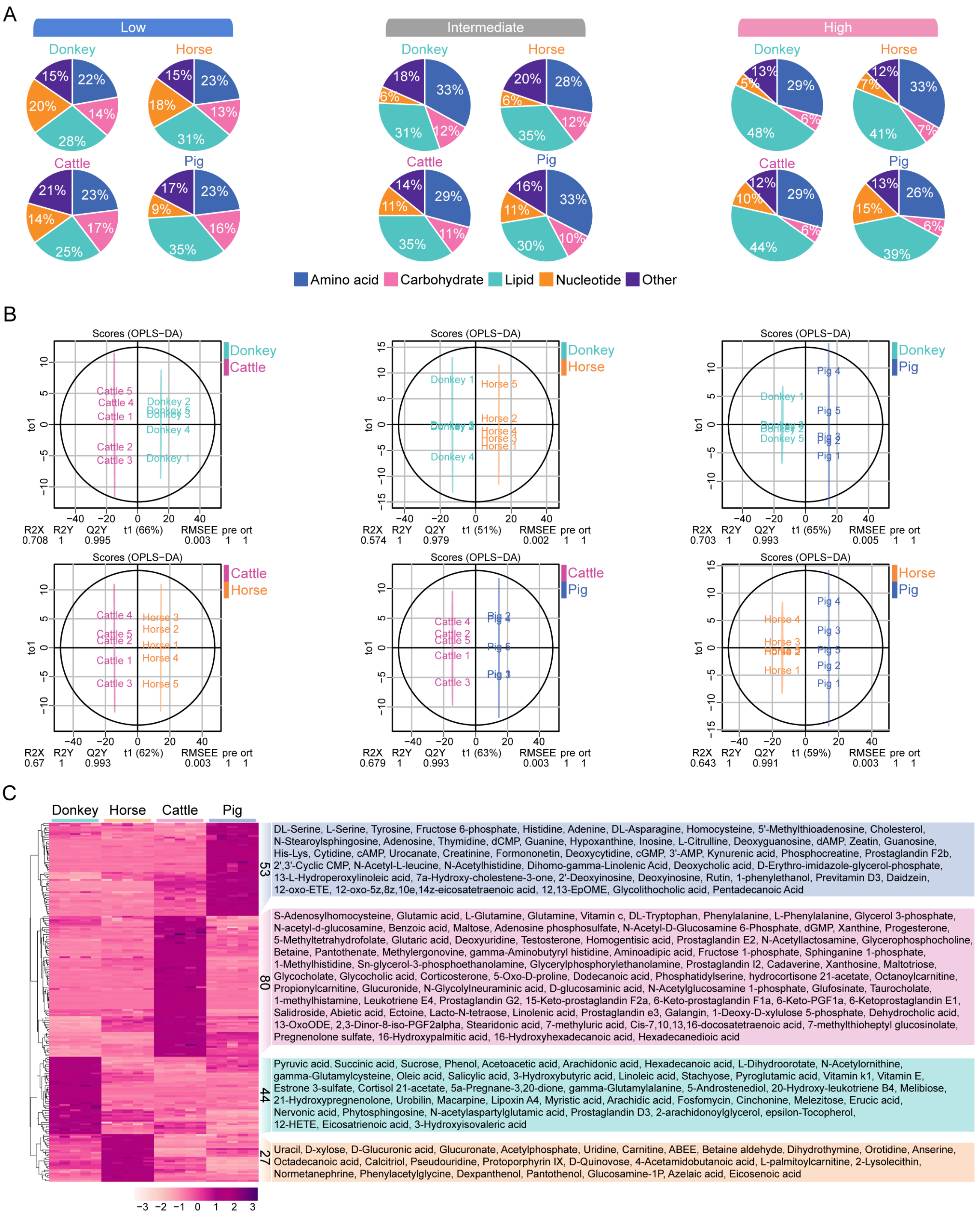

Figure S15

Metabolome differential analysis of donkey, horse, cattle, and pig skin. A) The pie charts illustrate the proportion of metabolite classes at high, medium, and low abundance levels in the skin of the four species. B) OPLS-DA score plot for discriminating the donkey, horse, cattle and pig skin metabolome. C) Heatmap showing the relative abundance of highly abundant

metabolites specific to donkey, horse, cattle, and pig skin.

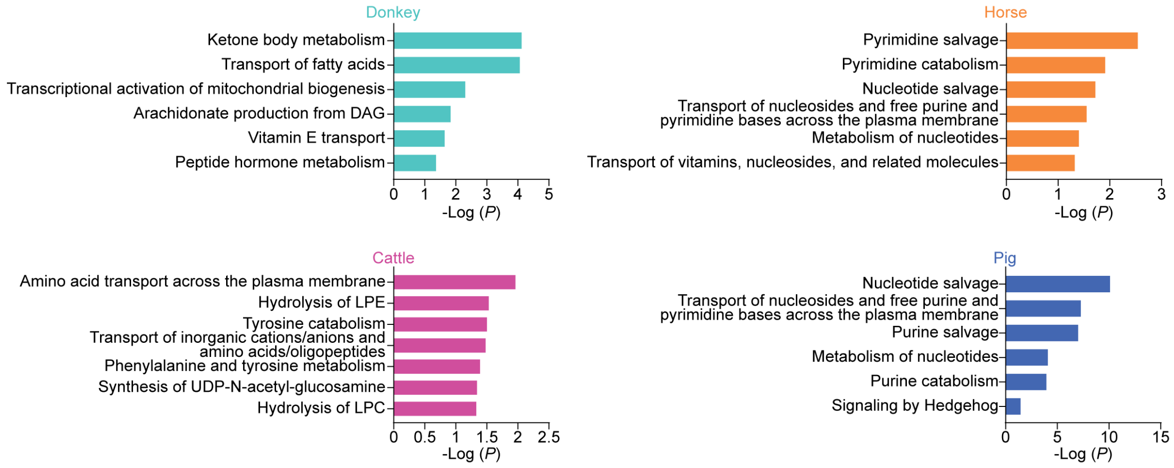

Figure S16

Reactome analysis of four species-specific high-abundance metabolites.

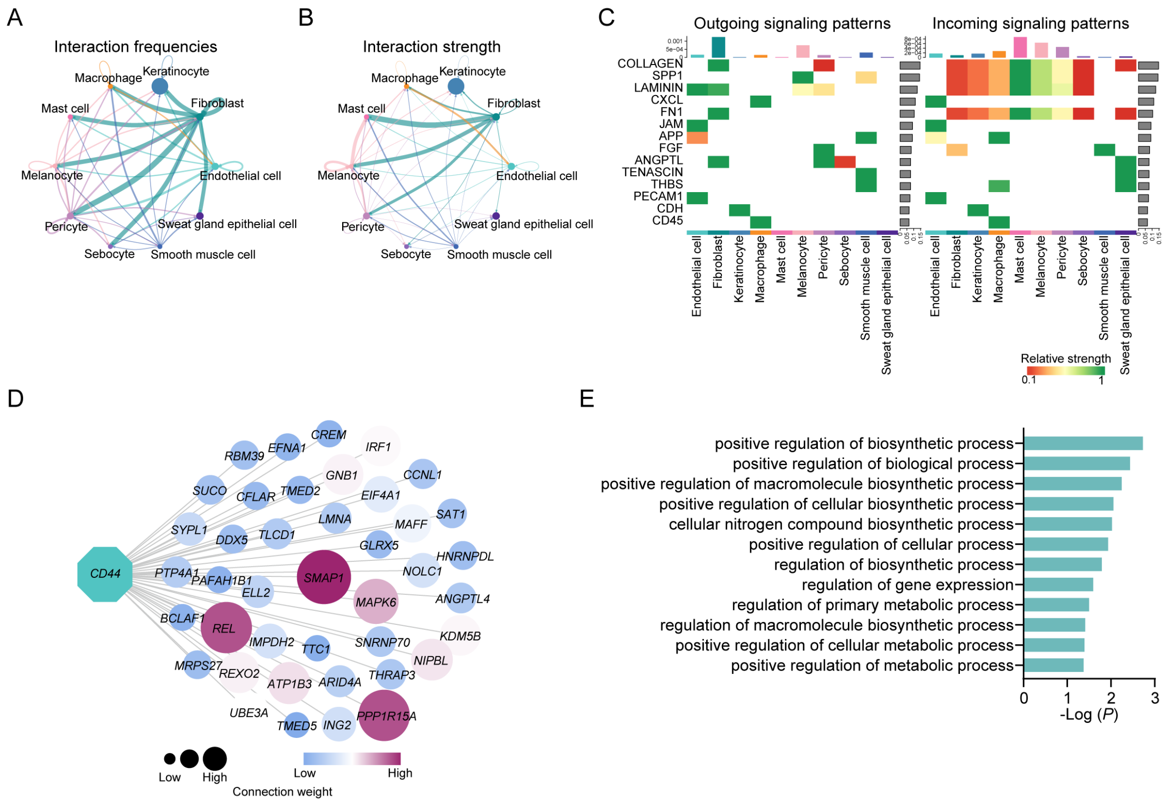

Figure S17

Analysis of cell-cell communication in donkey skin. A) The circos plots illustrate the frequency of interactions among major cell types within the donkey skin. B) The circos plots illustrate the strength of interactions among major cell types within the donkey skin. C) Heatmap illustrating the incoming and outgoing signal strength for each cell type in donkey skin. D) Network diagram showing CD44 regulated target genes. The node size indicates the weight of a connection. E) GO enrichment analysis results of CD44-regulated target genes.
